# Integrating Pharmacogenomics Data-Driven Computational Drug Prediction with Single-Cell RNAseq to Demonstrate the Efficacy of a NAMPT Inhibitor against Aggressive, Taxane-Resistant, and Stem-like Cells in Lethal Prostate Cancer

**DOI:** 10.3390/cancers14236009

**Published:** 2022-12-06

**Authors:** Suman Mazumder, Taraswi Mitra Ghosh, Ujjal K. Mukherjee, Sayak Chakravarti, Farshad Amiri, Razan S. Waliagha, Farnaz Hemmati, Panagiotis Mistriotis, Salsabil Ahmed, Isra Elhussin, Ahmad-Bin Salam, Windy Dean-Colomb, Clayton Yates, Robert D. Arnold, Amit K. Mitra

**Affiliations:** 1 Department of Drug Discovery and Development, Harrison College of Pharmacy, Auburn University, Auburn, AL 36849, USA; 2 Center for Pharmacogenomics and Single-Cell Omics (AUPharmGx), Harrison College of Pharmacy, Auburn University, Auburn, AL 36849, USA; 3 Department of Urology Research, Brigham and Women’s Hospital, Harvard Medical School, Boston, MA 02215, USA; 4 Department of Business Administration, University of Illinois at Urbana-Champaign, Champaign, IL 61820, USA; 5 Biomedical and Translational Sciences, Carle Illinois College of Medicine, University of Illinois Urbana-Champaign, Champaign, IL 61820, USA; 6 Department of Chemical Engineering, Samuel Ginn College of Engineering, Auburn University, Auburn, AL 36849, USA; 7 Department of Biology and Canter for Cancer Research, Tuskegee University, Tuskegee, AL 36088, USA; 8 Piedmont Hospital, Newnan, GA 30309, USA; 9 Department of Pathology, Johns Hopkins University School of Medicine, Baltimore, MD 21218, USA; 10 Sidney Kimmel Comprehensive Cancer Center, Johns Hopkins University School of Medicine, Baltimore, MD 21218, USA; 11 Department of Urology, Johns Hopkins University School of Medicine, Baltimore, MD 21218, USA; 12 UAB O’Neal Comprehensive Cancer, University of Alabama at Birmingham School of Medicine, Birmingham, AL 35233, USA

**Keywords:** secDrug, FK866, taxane, prostate cancer, synergism, RNAseq, scRNAseq, DEGs, IPA

## Abstract

**Simple Summary:**

Prostate cancer (PCa) is the second most common cancer and the second leading cause of cancer deaths in US men. Resistance to standard medical castration and secondary taxane-based chemotherapy, the presence of cancer stem-like cells representing epithelial to mesenchymal transdifferentiation (EMT), and neuroendocrine (NEPC) subtypes are serious causes of concern for prostate cancer (PCa) treatment. Drug development against these advanced/lethal variants of PCa is, therefore, a significant unmet challenge. We have designed a novel computational prediction algorithm called “secDrug” that identified novel secondary drugs for the management of advanced-stage cancers. Using FK866 (a nicotinamide phosphoribosyltransferase/NAMPT inhibitor) as a proof-of-concept secDrug, we established a novel, universally applicable, preclinical drug development pipeline that incorporates bulk-tumor and single-cell RNA sequencing, microfluidics, as well as in vitro (cell line models representing clinically advanced PCa), and patient-based validation to introduce secondary drug choices to potentially circumvent subclonal aggressiveness, drug resistance, and stemness for the management of lethal subtypes of PCa.

**Abstract:**

Metastatic prostate cancer/PCa is the second leading cause of cancer deaths in US men. Most early-stage PCa are dependent on overexpression of the androgen receptor (AR) and, therefore, androgen deprivation therapies/ADT-sensitive. However, eventual resistance to standard medical castration (AR-inhibitors) and secondary chemotherapies (taxanes) is nearly universal. Further, the presence of cancer stem-like cells (EMT/epithelial-to-mesenchymal transdifferentiation) and neuroendocrine PCa (NEPC) subtypes significantly contribute to aggressive/lethal/advanced variants of PCa (AVPC). In this study, we introduced a pharmacogenomics data-driven optimization-regularization-based computational prediction algorithm (“secDrugs”) to predict novel drugs against lethal PCa. Integrating secDrug with single-cell RNA-sequencing/scRNAseq as a ‘Double-Hit’ drug screening tool, we demonstrated that single-cells representing drug-resistant and stem-cell-like cells showed high expression of the NAMPT pathway genes, indicating potential efficacy of the secDrug FK866 which targets NAMPT. Next, using several cell-based assays, we showed substantial impact of FK866 on clinically advanced PCa as a single agent and in combination with taxanes or AR-inhibitors. Bulk-RNAseq and scRNAseq revealed that, in addition to NAMPT inhibition, FK866 regulates tumor metastasis, cell migration, invasion, DNA repair machinery, redox homeostasis, autophagy, as well as cancer stemness–related genes, HES1 and CD44. Further, we combined a microfluidic chip-based cell migration assay with a traditional cell migration/‘scratch’ assay and demonstrated that FK866 reduces cancer cell invasion and motility, indicating abrogation of metastasis. Finally, using PCa patient datasets, we showed that FK866 is potentially capable of reversing the expression of several genes associated with biochemical recurrence, including IFITM3 and LTB4R. Thus, using FK866 as a proof-of-concept candidate for drug repurposing, we introduced a novel, universally applicable preclinical drug development pipeline to circumvent subclonal aggressiveness, drug resistance, and stemness in lethal PCa.

## 1. Introduction

Prostate cancer (PCa) is the second leading cause of non-cutaneous cancer-related deaths in US men [1,2]. The androgen signaling pathway plays a crucial role in PCa development [3,4]. Most prostate cancers are androgen-sensitive malignancies whose growths depend on overexpression of the androgen receptor (AR). Therefore, Androgen deprivation therapies (ADT), including surgical castration, medical castration, anti-androgens like enzalutamide, and androgen biosynthesis inhibitors, are the standard first-line treatment for PCa [3,5,6]. Most of the early-stage PCa patients who have been treated with ADT show good initial responses. However, a vast majority of men with early-stage PCa eventually become unresponsive to these primary treatment options, and despite low levels of androgen, the disease progresses with continuously rising Prostate Serum Antigen (PSA), ultimately developing more aggressive forms called Castration-resistant prostate cancer (CRPC) [7,8,9,10]. Metastatic castration-resistant prostate cancer (mCRPC) is the clinically most advanced and lethal disease state with signs of metastasis to distant organs like the brain, bone, lung, and lymph nodes and it has a median survival of fewer than three years (5-year median survival rate of 31%) [11,12]. Although next-generation AR-targeting chemotherapeutic treatments like abiraterone plus prednisone (AA/P) or enzalutamide (ENZ), and in combination with taxanes (Docetaxel or DTX), increase the survival rate slightly, eventual development of resistance (acquired resistance) is nearly universal where progression-free survival approaches ~0% in 3 years, often with severe side effects [11,13,14,15,16,17,18,19,20,21]. Chemotherapy options become limited once patients fail DTX therapy. Moreover, variable drug response observed among patients (only ≈50% of men show measurable DTX response) due to innate or refractory resistance is also a cause of concern in mCRPC chemotherapy [15,16]. Treatment with the second-generation anti-mitotic taxane, Cabazitaxel (CBZ), in patients who have received prior DTX confers little improvement (≈2.4 months) in the median OS and causes severe side effects [17]. Further, neuroendocrine PCa or NEPC (also known as small cell carcinoma) is an intrinsically resistant, poorly differentiated aggressive variant of PCa that lacks AR expression [22,23]. In addition, several groups, including ours, have shown that the presence of cancer stem-like cells (CSCs) like side populations (SPs) and CD133^+^ cells with self-renewal and differentiation (acquisition of mesenchymal phenotype or epithelial to mesenchymal transdifferentiation/EMT) significantly contribute to tumor aggressiveness and the development of drug resistance [24,25,26].

Drug development for treating these lethal/aggressive/advanced variants of PCa (AVPC) thus poses a significant challenge with very few therapeutic successes.

Our laboratory has designed a pharmacogenomics data-driven computational pipeline (secDrug) that identifies novel secondary drugs (“secDrugs”) for the treatment of drug-resistant advanced-state cancers [27].

In this study, we applied the secDrug algorithm to prostate cancer and identified several novel secondary drug candidates for the treatment of AVPC. Next, using single-cell RNA sequencing (scRNAseq), we demonstrated the presence of PCa subclones representing aggressive, TX-resistant, and cancer stem-like cells. Further, our scRNAseq data predicted that the secDrug, FK866 (a NAMPT inhibitor), is potentially effective against PCa subclones with the enrichment of treatment-resistant and stem-like genes. We hypothesize that our predicted and pre-screened secDrugs would be helpful in curbing oncogenic progressions as a single agent or in combination with taxanes in AVPC through simultaneous inhibition of multiple oncogenic factors/pathways. Using in vitro model systems of treatment-refractory and treatment-emergent AVPC (representing mCRPC, NEPC, and EMT), we demonstrated that the FK866 not only showed efficacy as a single-agent but also enhanced the efficacy of the taxane drugs DTX and CBZ. Further, we performed a sophisticated microfluidic chip-based confined cell migration assay that recapitulates diverse micro-environmental cues encountered by cancer cells during locomotion (e.g., the dimensionality of pores and 3D longitudinal, channel-like tracks) to investigate the effect of FK866-based regimens on cancer cell invasion, motility, and metastasis. Finally, we demonstrated the impact of FK866 in eroding ‘stem-like’ subpopulations (including SPs, quiescent/dormant cells, and ALDH1+cells).

Earlier studies have shown that FK866 inhibits the growth of PCa-engineered tumors by interfering with energy metabolism [28,29]. However, the clinical benefit and comprehensive mechanism of action (MOA) for FK866 have not been fully uncovered. Therefore, we performed pre- vs. post-treatment bulk and single-cell tumor RNAseq to identify differentially expressed genes (DEGs) and potential molecular pathways associated with the FK866 mechanism of action in AVPC at the tumor and subclonal levels. Finally, using comparative analysis of whole-genome transcriptomics data between clinically sensitive and resistant PCa patients, we demonstrated that FK866 has the potential to be clinically effective based on the reverse matching of GEP signatures and top dysregulated pathways.

## 2. Materials and Methods

### 2.1. Drugs and Reagents

Drugs, reagents, antibodies, and kits are listed in Supplementary Table S1.

### 2.2. Human Prostate Cancer Cell Lines

AR^lo^ mCRPC/NEPC (PC3, PC3M, DU145) and AR^high^ mCSPC (LnCAP, 22Rv1) cell lines, and the LnCAP-derived Androgen-independent, osteotropic subline C42b were obtained from the American Type Culture Collection (ATCC) (Manassas, VA, USA). The taxane-resistant cell lines PC3-TXR and DUTXR were generated using dose-escalation of taxanes over time, as described earlier [30]. The cell lines were authenticated at the source and tested randomly at regular intervals for tissue specimen provenance and cell lineage at the AU Center for Pharmacogenomics and Single-Cell Omics (AUPharmGx) using GenePrint 24 System (Promega). All cell lines are mycoplasma negative. PC-3, PC-3M cells were maintained in 10% (*v*/*v*) (FBS) supplemented in F-12K, DU145 in Eagle’s Minimum Essential Medium (EMEM). PC3-TXR and DUTXR were maintained in RPMI-1640 media with 1% Penicillin-Streptomycin at 37 °C, 21% O_2_, and 5% CO_2_ in a humidified cell culture chamber (Heracell™ VIOS 160i CO_2_; Thermo Fisher Scientific, Waltham, MA, USA).

### 2.3. Patient Datasets

Next-generation RNAseq data on PCa patient samples at the Atlanta VA Medical Center, Decatur, Georgia, Moffitt Cancer Center, (Tampa, FL, USA) and the Sunnybrook Health Science Center (Toronto, ON, Canada) were obtained from the Gene expression omnibus database (GSE54460). Details on patient samples have been provided earlier [31]. Briefly, FFPE tissue blocks were obtained, following Institutional Review Board approvals, from patients at the Atlanta VA Medical Center, Decatur, Georgia, Moffitt Cancer Center, Tampa, FL, USA) and the Sunnybrook Health Science Center (Toronto, ON, Canada) and next-generation RNAseq was performed on 100 patients. Demographic information (age, race), cancer staging (Gleason score, pSpTage), and biochemical recurrence was available for 100 patients. Among these cases, 49 had biochemical recurrence (BCR), and 51 had no BCR [31]. 

Gene expression on PCa patients included in The Cancer Genome Atlas (TCGA) database was extracted from the Genomic Data Commons (GDCs) server (cancergenome.nih.gov). The interactive web-portals UALCAN and Gene Expression Profiling Interactive Analysis (GEPIA) were used for in-depth analysis of TCGA gene expression data files and to compare transcriptome data on target candidate pathway genes with tumor metastasis and patient survival from the prostate expression data matrix [32,33].

### 2.4. Identification of Secondary Drugs (secDrugs) in Lethal Prostate Cancer

The design and development of the secDrug algorithm have been described earlier [27]. Briefly, we used a pharmacogenomics data-driven approach to identify potential agents that can be re-purposed as novel secondary drugs to treat cancers resistant to standard-of-care (primary) drugs when used in combination with the primary drug. As the data source, we used the GDSC1000 (Genomics of Drug Sensitivity in Cancer) database, a large-scale pharmacogenomics database of dose-response results (IC_50_ or AUC) on 265 compounds in >1000 cell lines representing a wide spectrum of human cancers [34]. These 265 drugs cover a wide range of targets and processes involved in cancer biology, which include drugs that are either approved and used in the clinic, or are undergoing clinical development, or are in clinical trials, or are tool compounds in early-phase development. For the purpose of this study, to predict secDrugs in Prostate Cancer, we used an inclusion criterion that filtered cell lines with genito-urinal cancer subtypes. A total of 136 cell lines were selected from the GDSC1000 database breast (*n* = 52), cervix (*n* = 14), endometrium (*n* = 11), ovary (*n* = 45), prostate (*n* = 8), testis (*n* = 3), and vulva (*n* = 3). 

First, we assumed that IC_50_ values of DTX in these lines (including PCa cell lines) were$\;S_{bi}:i\in \left\{1,\cdots ,n\right\}$, where there are n cell lines. In addition, we assumed that there are K other drugs, and the IC_50_ values of the n cell lines for the K drugs are given by:

(1) $$R_{ki}:k\in \left\{1,\cdots ,K\right\},\;i\in \left\{1,\cdots ,n\right\}.$$

Next, we classified the cell lines as sensitive or resistant to DTX using a quantile of the empirical distribution of $R_{ki}$, and a threshold criterion to achieve the classification. Finally, we identified secondary drugs or secDrugs that could kill the maximum number of DTX-resistant cell lines based on individual IC_50_ values. In the case of ties between the top secDrugs, we chose the drug with the lower mean IC_50_ values.

### 2.5. In Vitro Cytotoxicity Assays and Drug Synergy Analysis 

In vitro chemo-sensitivity assays were performed on human PCa cell lines using mitochondrial enzyme activity or MTT (3-(4,5-dimethylthiazol-2-yl)-2,5-diphenyltetrazolium bromide reagent) assay. Briefly, cells were plated in a 96-well culture plate at 2 × 10^3^ cells/well and incubated for 24 h at 37 °C with 5% CO_2_. Cells were treated with increasing concentrations of DTX (0–2250 nM), CBZ (0–2250 nM), Enzalutamide (0–5062.5 nM), Bicalutamide (0–5062.5 nM), and FK866 (0–625 nM) as a single agent, or the combination of DTX + FK866, and CBZ + FK866. For mCSPC lines, cells were also treated with Enzalutamide (ENZ) single-agent and a ENZ + FK866 combination. Following 48-h of incubation, the tetrazolium dye MTT was added according to the manufacturer’s instructions, and absorbance was measured at 550 nm using Synergy Neo2 Microplate Reader (BioTek, Santa Clara, CA, USA). Percent change relative to untreated controls was calculated at each drug concentration, and the effect of drug exposure was determined by constructing cytotoxicity (growth) curves. Half-maximal inhibitory drug concentration (IC_50_) values were estimated by nonlinear regression using a sigmoidal dose-response equation (variable slope). Drug synergy was calculated by comparing single-agent and combination drug-response data based on Chou-Talalay’s combination index (CI) method and the isobologram algorithm (CompuSyn software; Biosoft, US) [35]. CI values between 0.9–0.3 and 0.3–0.1 signify synergism and strong synergism, respectively, between the drugs treated in combination. 

### 2.6. Caspase-3/7 Activity Assay 

Cell death by apoptosis was measured using the Caspase-Glo 3/7 luminescent assay system kit according to the manufacturer’s instructions (Promega Madison, WI, USA). Briefly, 2 × 10^3^ cells/well were seeded into 96-well plates (triplicates) and treated at the estimated single-agent vs. combination IC_50_ values calculated by the MTT assay. Following 48 h of incubation, Caspase-Glo 3/7 reagent was added, incubated for 2 h, and luminescence was measured using a Synergy Neo2 Microplate Reader (BioTek, USA). The apoptosis level in each treatment group was normalized to the control group (no drug treatment with baseline caspase 3/7 assay luminescence) for each cell line.

### 2.7. Annexin V and Propidium Iodide (PI) Staining

Annexin V and PI staining was used to assess apoptosis and necrosis by flow cytometry. Briefly, cells were seeded in 6-well plates at indicated concentrations and exposed to DTX and FK866 as a single agent and as combinations. After 48 h, cells were labeled with a binding buffer containing annexin V-FITC (25 µg/mL) and PI (25 µg/mL) as well as 10 mM HEPES, 140 mM NaCl, 5 mM KCl, 1 mM MgCl_2_, and 1.8 mM CaCl_2_ (pH = 7.4), incubated for 10 min, followed by three washes in the binding buffer. Both detached and attached cells were combined, and staining was quantified using a Becton Dickinson FACS Calibur flow cytometer (BD Biosciences, San Jose, CA, USA) at 10,000 events per measurement.

### 2.8. Assessment of Cellular and Nuclear Morphology

To assess cellular morphology, PCa cells were seeded 0.025 × 10^6^ cells/mL in 6-well plates and exposed to FK866, either as a single agent or in combination with DTX for 48 h. Three areas with approximately equal cell densities were identified in each well, and images were captured with an EVOS FL digital cell imaging system (Thermo Fisher Scientific, Waltham, MA, USA) using a 10X objective. 

For nuclear morphology, PCa cells were plated on top of the glass coverslip (1.5 × 10^5^ cells/mL), incubated overnight, and treated with either vehicle or FK866 alone or as a combination with DTX. After 48 h, the cells were labeled with NucBlue Live reagent and incubated for 20 min. Images were captured using Nikon Eclipse Ti2 microscope and recorded in a bright field and phase contrast modes were at 20X and 40X magnifications. Images were analyzed using Image J software v 1.53 (National Institutes of Health, Bethesda, MD, USA).

### 2.9. NAD/NADH Activity Assay

PCa cell lines (PC-3, PC-3M, DU145, and DUTXR) were plated in a white-welled luminometer 96-well plate at a seeding density of 2 × 10^3^ cells/well and treated with indicated concentrations of FK866 as a single agent. Following 48 h of incubation, NAD cycling enzyme, NAD Cycling Substrate, and NAD/NADH-Glo Detection Reagent were added to the cells and incubated for 1 h. Luminescence was measured using a Synergy Neo2 plate reader (BioTek, USA). 

### 2.10. Assessment of Cell Cycle

Control (no drug) and post-treated cells were prepared for cell cycle analysis by staining with PI (50 µg/mL) in a sample buffer [PBS + 1% (*w*/*v*) glucose], containing RNAse A (100 units/mL) for 30 min at room temperature and analyzed by flow cytometry using a Becton Dickinson FACS Calibur flow cytometer (BD Biosciences, San Jose, CA, USA). Cell cycle data were analyzed using CytExpert (Beckman Coulter Inc., Indianapolis, IN, USA). Data are presented as the mean ± SEM of three separate experiments (*n* = 3/study).

### 2.11. Assessment of Intracellular ROS Levels (DCFDA Assay)

Cells were plated at a seeding density of 2000 cells/well and incubated overnight at 37 °C. After 24 h, 100 uL of 10 uM DCFDA solution was added to each well and incubated in the dark for 45 min at 37 °C. The DCFDA solution was then discarded and treated with either a vehicle (0.5% DMSO) or the FK866 single agent and in combination with DTX. Samples were collected at different time points (2, 4, 8, and 24 h). Fluorescent intensity was measured on Synergy Neo2 Hybrid Multi-Mode Microplate Reader, BioTek (Winooski, VT, USA) at excitation—485 nM and emission—535 nm in endpoint mode. 

### 2.12. Assessment of Mitochondrial Membrane Potential

Cell lines were treated with DTX, FK866, and DTX + FK866. Further, 100 μL/well of working JC-1 solution was added to the plate and incubated at 37 °C for 10 min in the dark. Read plate endpoint in the presence of compounds, and media on a fluorescent plate reader Synergy Neo2 Hybrid Multi-Mode Microplate Reader, BioTek (Winooski, VT, USA) at 535 nm. 

### 2.13. Assessment of Side Population

A total of 1 × 10^6^ /mL cells were cultured in 6-well plates and treated with FK866 alone or in combination with DTX. After 24 h, cells were stained with 5 μM Vybrant DyeCycle Violet and 1 ug of 7-AAD for 30 min at 37 °C. Following dye incubation, cells were immediately analyzed (10,000 events per measurement) using a Becton Dickinson FACS Calibur flow cytometer (BD Biosciences, San Jose, CA, USA).

### 2.14. Colony Formation Assay

PCa cells were seeded in a 6-well plate at 0.025 × 10^6^ cells/mL, incubated overnight_,_ and treated with DTX and FK866 as a single agent or in combination. The cells were then harvested and plated in a 24-well plate at a concentration of 1000 cells/well and incubated for 1–2 weeks. The colonies were fixed with 100% methanol and stained with Crystal Violet. Images were taken for the control, the treated cells, and the colonies using an EVOS FL digital cell imaging system (ThermoFisher Scientific, Waltham, MA, USA). Images were recorded in a bright field and the phase contrast modes were at 20X and 40X magnifications and analyzed using Image J software.

### 2.15. Microfluidic (μ)-Channel Cell Migration Assay

The fabrication of a Polydimethylsiloxane (PDMS)-based μ-channel assay using standard multilayer photolithography and replica molding has been demonstrated earlier (Figure S3) [36]. In this study, PCa cells were seeded in 6-well plates exposed to FK866 as a single agent and FK866 + DTX combinations at indicated concentrations. Next, 1–1.5 × 10^5^ cells were introduced into the cell seeding inlet line of the microfluidic channel via a pressure-driven flow and were allowed to adhere for 30 min at 37 °C, 5% CO_2_. Next, the cell suspension was removed and substituted with a serum-free medium. The medium, supplemented with 10% FBS, was added into the chemoattractant inlet line to trigger cell entry into the channels. The devices were placed on an automated Nikon Ti2 Inverted Microscope equipped with a Tokai Stage-Top incubator unit, which maintained cells at 37 °C and 5% CO_2_. Cell entry into the channels was recorded via time-lapse microscopy. Images were recorded every 20 min for 10 h with a 10×/0.45 NA Ph1 objective.

### 2.16. Cell Migration/Scratch Assay

Cells were plated in 6-well plates at 1 × 10^5^ cells/well and incubated for 48 h to a 95% confluency. The monolayer was scratched with a SPLScar Scratcher 6-well Tip at a width of 0.50 mm at the center of the well. FK866 as single-agent or DTX + FK866 combination doses were applied to the cells in the respective wells. The F-12K culture medium, supplemented with 10% FBS and containing the vehicle (0.05% DMSO) was added to the cells in the control wells. Micrographs of the wound areas were obtained at 0, 24, and 48 h using an EVOS FL digital cell imaging system (Thermo Fisher Scientific, Inc.). Images were recorded in a bright field and phase contrast modes were at 20X and 40X magnifications. The area of the initial wound (at 0 h) and the “gap area” were measured at 48 h with Image J software.

### 2.17. Pre- and Post-Treatment Tumor mRNA Sequencing (RNAseq)

The effects of DTX and FK866 as a single agent and in combination and its exposure on gene expression in PCa cell lines were assessed using next-generation RNAseq of bulk tumor cells. Pre- and post- drug-exposure (FK866 single-agent, TX + FK866 combination) tumor cells were harvested, and high-quality RNA was extracted using QIAshredder and the RNeasy kit (Qiagen) according to the manufacturer’s protocol. RNA concentration and integrity were assessed using the Nanodrop-8000 spectrophotometer (Thermo Fisher Scientific, Wilmington, DE, USA), Qubit 2.0 Fluorometer (Invitrogen, Carlsbad, CA, USA), and Agilent 2100 Bioanalyzer (Applied Biosystems, Carlsbad, CA, USA) and stored at −80 °C. An RNA integrity number (RIN) threshold >8 was applied, and RNAseq libraries were constructed using the Illumina TruSeq RNA Sample Preparation kit v2. Libraries were then size-selected to generate inserts of ~200 bp, and RNAseq was performed on Illumina’s NovaSeq platform using a 150 bp paired-end protocol with a depth of >20 million reads per sample. Average quality scores were thoroughly above Q30 for all libraries in both R1 and R2.

RNAseq data analysis: RNAseq data from the cell lines and patient RNAseq data (described above) was pre-processed and normalized, and a differential expression (DE) analysis was performed using a command-line based analysis pipeline (DEseq2 and edgeR) and the Partek Flow software (Partek, Inc., St. Louis, MO, USA). Quality control (QC) check on the RNAseq raw reads was performed using the FastQC tool, followed by read trimming to remove base positions that have a low median (or bottom quartile) score. STAR Aligner tool mapped processed RNAseq reads to the hg38 human genome build. Next, read counts were CPM-normalized, and then we used GSA (Gene-specific analysis) based on a limma trend that applies an empirical Bayesian method to perform differential gene expression analysis between groups and detect the DE genes. Genes with a mean fold-change >|1| and *p* < 0.05 were considered as the threshold for reporting significant differential gene expression. Heatmaps were generated using an unsupervised hierarchical clustering (HC) analysis based on the differentially expressed genes (DEGs). 

### 2.18. Pre- and Post-Treatment Single-Cell RNA Sequencing (scRNAseq)

Automated single-cell capture and cDNA synthesis were performed on the untreated and FK866-treated acquired taxane-resistant mCRPC DUTXR using the 10X Genomics Chromium platform. Single-cell RNAseq-based gene expression analysis will be performed on Illumina HiSeq 2500 NGS platform (Paired-end. 2 × 125 bp, 100 cycles. v3 chemistry) at ~10 million reads per sample.

ScRNAseq data analysis: Single-cell RNAseq datasets were obtained as matrices in the Hierarchical Data Format (HDF5 or H5). We used CellRanger, Seurat, and Partek Flow software packages to pre-process the scRNAseq data and perform single-cell transcriptomics. Highly variable genes were selected for the clustering analysis based on a graph-based clustering approach. The visualization of cell populations was performed by T-distributed stochastic neighbor embedding (t-SNE) and UMAP (Uniform Manifold Approximation and Projection) for a biomarker-based identification of subclones representing TX-resistant cells, potential FK866 target subclones, and cancer stem-cell signatures, as well as FK866 treatment-induced erosion of these subclones.

### 2.19. Ingenuity Pathway Analysis (IPA)

Ingenuity pathway analysis (IPA; Qiagen, Redwood City, CA, USA) was performed using top DEGs to reveal molecular pathways/mechanisms, upstream regulator molecules, downstream effects, biological processes, and predicted causal networks governing FK866 function and successful drug combinations in AVPC [37].

### 2.20. Immunoblotting

PCa cells were seeded and exposed to drugs at the indicated concentrations of each treatment protocol. Post-treatment cells were lysed in a cell lysis buffer (Thermo Scientific RIPA Lysis and Extraction Buffer). Quantification of proteins was performed using Bradford assay, and a calibration curve of protein content was created from the BSA protein standard kit (Bio-Rad Laboratories, Hercules, CA, USA). An equal amount of protein (50 ng) was loaded onto 4–15% Criterion TGX Stain-Free Precast Gels. Proteins were separated under reducing conditions and then transferred to a PVDF membrane using the Fisherbrand Semidry Blotting Apparatus. Nonspecific binding was limited by incubating the membrane in a blocking buffer (2.5% (*w*/*v*) casein, pH 7.6, 150 mM NaCl, 10 mM TRIS-HCl, and 0.02% sodium azide). Membranes were incubated overnight with primary antibodies for the targeted gene/protein (1:1000) and then with the appropriate secondary antibody (1:10,000) for 1.5 h at room temperature. Immunoreactivity was detected using the Pierce ECL Western Blotting substrate (Bio-Rad, Hercules, CA, USA). Images were captured and quantified by the Gel Doc EZ Gel Documentation System and ImageLab Software (Hercules, CA, USA). Densitometry analysis was performed using the standard image analysis software Image J.

### 2.21. Statistical Analysis 

All statistical analysis was performed using R (the project for statistical computing and graphics) version 4.1.0 and GraphPad Prism v9.0. All tests were two-sided, and *p* < 0.05 was considered statistically significant. We used a non-parametric Wilcoxon rank-sum test for differential expression analysis between two groups of cells. 

## 3. Results

### 3.1. Single Cell Transcriptomics (scRNAseq) Showed AR^low^ PCa Cells with Signatures of Epithelial-Mesenchymal Transition (EMT) and Cancer ‘Stemness’

**Figure 1:**
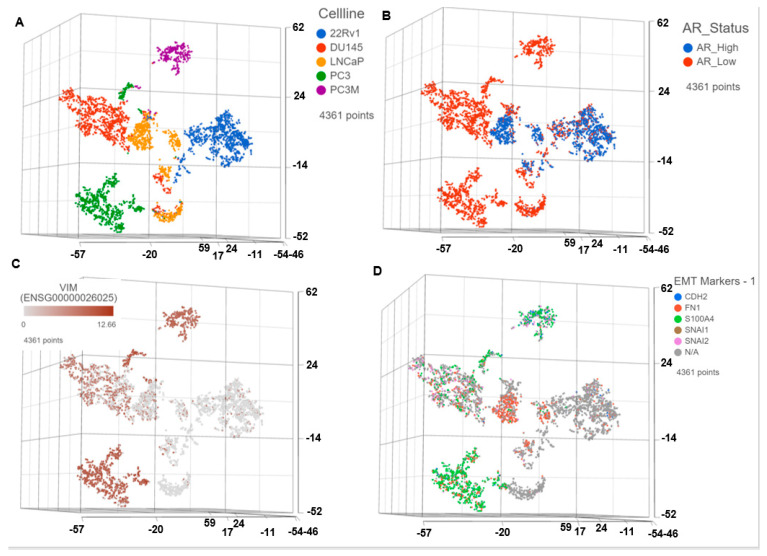

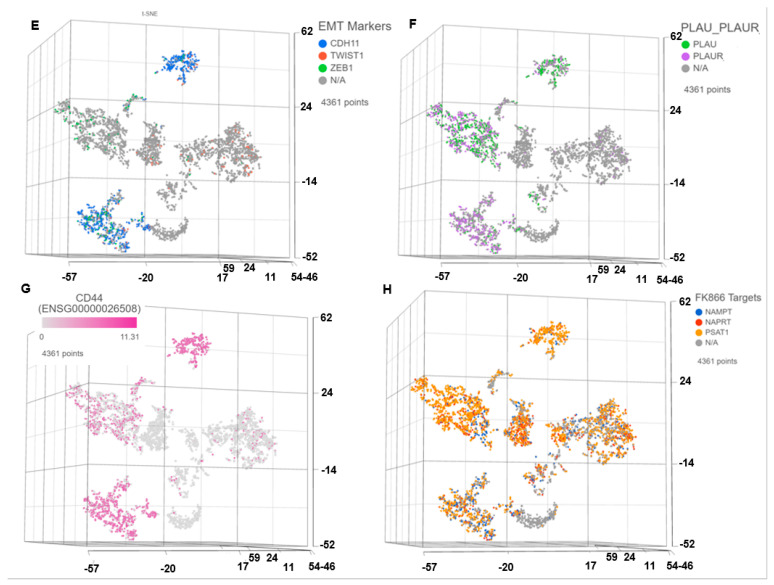
Single-cell transcriptomics identifies signatures of epithelial to mesenchymal transition (EMT) and stemness in metastatic prostate cancer cells. Single-cell RNA sequencing using the Droplet sequencing method (10X Genomics) was performed on the PCa cell lines 22Rv1, LnCAP, DU145, PC3, and PC3M. t-distributed stochastic neighbor embedding (tSNE) plots showing the comparison between the single-cell clusters representing (**A**) All cell lines; (**B**) AR status; Expression of mesenchymal markers involved in EMT transdifferentiation, including the (**C**) Vimentin (VIM); (**D**) N-Cadherin (CDH1), Fibronectin (FN1), S100A4, Snail (SNAI1), Slug (SNAI2); other major EMT markers (**E**) CDH11, TWIST1, ZEB1. Expression of genes potentially involved in Cancer stemness (**F**) Urokinase-type plasminogen activator (PLAU) and Urokinase-type plasminogen activator receptor (PLAUR); (**G**) CD44; FK866 target pathway genes (**H**) Nicotinamide phosphoribosyltransferase (NAMPT), Nicotinate Phosphoribosyltransferase (NAPRT), and Phosphoserine Aminotransferase 1 (PSAT1). Each dot represents a single cell. Contaminated (doublet) cells were not included.

To determine the transcriptome profiles of individual cells, we performed single-cell sequencing of mCSPC and mCRPC cells. Figure 1A displays t-SNE clusters generated from baseline (untreated) scRNAseq data in mCSPC and mCRPC cell lines. Each dot represents a single cell. Further, the AR status of each cell is represented in Figure 1B. Epithelial-mesenchymal transitions have been mechanistically linked with the generation and maintenance of stem-like cell populations during tumorigenesis. PCa cells that have undergone EMT are phenotypically and genomically similar to stem cells. For example, Vimentin is a well-characterized filament protein that is highly expressed in mesenchymal cells. Thus, enhanced levels of Vimentin and downregulation of E-cadherin served as markers for identifying cells that have undergone EMT. Figure 1C–E demonstrates that the AR^low^ cells (primarily belonging to the mCRPC subtype) show a higher expression of several mesenchymal gene signatures involved in Epithelial-mesenchymal transition with NEPC phenotype, including Vimentin (Figure 1C); N-cadherin (CDH2), Fibronectin (FN1), S100A4, Snail (SNAI1), Slug (SNAI2) (Figure 1D); and other major EMT markers CDH11, TWIST1, ZEB1 (Figure 1E). Further, Figure 1F,G shows upregulation of cancer stemness-related markers Urokinase-type plasminogen activator (PLAU), Urokinase-type plasminogen activator receptor (PLAUR), and CD44, primarily in mCRPC cells. 

Interestingly, signatures of cancer stemness and EMT transdifferentiation were also observed in a subgroup of AR^low^ single cells within the mCSPC cell lines, 22Rv1, LnCAP.

**Figure 2:**
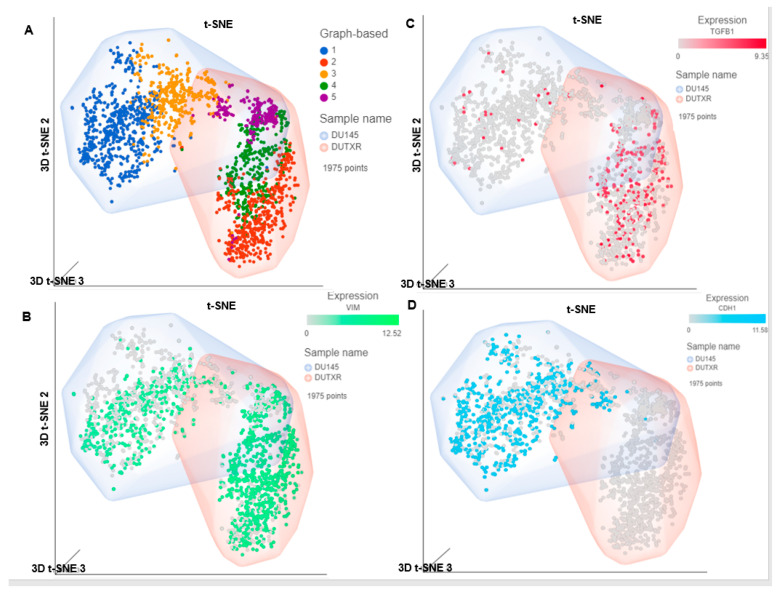

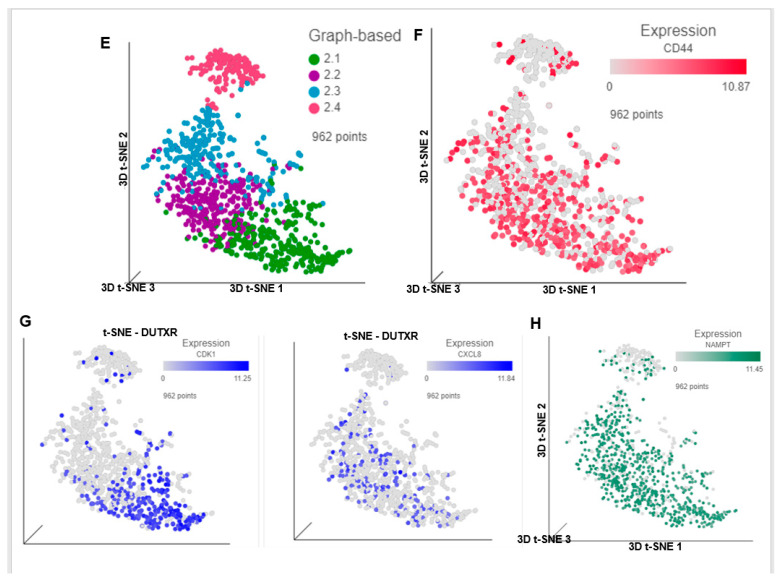
Comparison of single-cell RNAseq data between DU145 and DUTXR. (**A**) All 4 t-SNE clusters, (**B**–**D**) showing the EMT markers TGF-B1, VIM and E-Cadherin (CDH1). (**E**) scRNAseq data shows DUTXR cells representing enrichment of gene signatures of (**F**) Cancer stemness CD44^high^; (**G**) Drug resistance (CDK1^high^; CXCL8^high^), and the (**H**) FK866 target NAMPT^high^. Each dot represents a single cell. Contaminated (doublet) cells were not included.

Next, we compared the single-cell gene expression markers between taxane-sensitive (DU145) and the clonally derived acquired taxane-resistant mCRPC cell line DUTXR (Figure 2). We observed upregulation of gene signatures association with mesenchymal transition (VIM and TGFB1) and downregulation of the epithelial marker epithelial cadherin/E-cadherin (CDH1) in the DUTXR cell line compared to DU145 (Figure 2B–D). Further, the taxane-resistant DUTXR also showed enrichment of biomarkers that play significant roles in cancer progression, development, and maintenance of cancer stemness (CD44; Figure 2E,F) and drug resistance (CDK1, CXCL8; Figure 2G), indicating probable involvement of these genes in mCRPC development and progression [38]. 

### 3.2. Pharmacogenomics Data-Driven Algorithm and scRNAseq-Based ‘Double-Hit’ Screening Predicted FK866 as a Top secDrug Potentially Effective against Lethal PCa 

Our pharmacogenomics data-driven in silico prediction algorithm (described in the Methods section) identified several potential agents that can be re-purposed as novel secondary drugs (“secDrugs”; Table 1) to treat DTX-resistant prostate cancer when used as a single agent or in combination with the primary drug (taxanes). These include FK866 (NAMPT inhibitor), TAK715 (p38 MAPK inhibitor), YM155 (survivin inhibitor), MK-2206 (Akt1/Akt2/Akt3 inhibitor), LY317615 (PKCβ inhibitor), XAV939 (Wnt/β-catenin pathway inhibitor), RDEA119 (MEK1/2 inhibitor), and WZ3146 (mutant-selective irreversible inhibitor of EGFR (L858R)/EGFR (E746_A750)). Interestingly, using scRNAseq as a biomarker-based drug screen, we demonstrated that a majority of the single-cell subclones in mCRPC and mCSPC cell lines also showed significantly high expression of the NAMPT pathway genes, indicating that FK866, which targets NAMPT, is potentially effective against these taxane-resistant and stem-cell-like subpopulation clusters (Figure 1H and Figure 2H). 

### 3.3. FK866 Induced Loss of Viability in PCa Cell Lines as Single-Agent Treatment and Showed Synergy with Taxanes and AR Inhibitors

**Figure 3:**
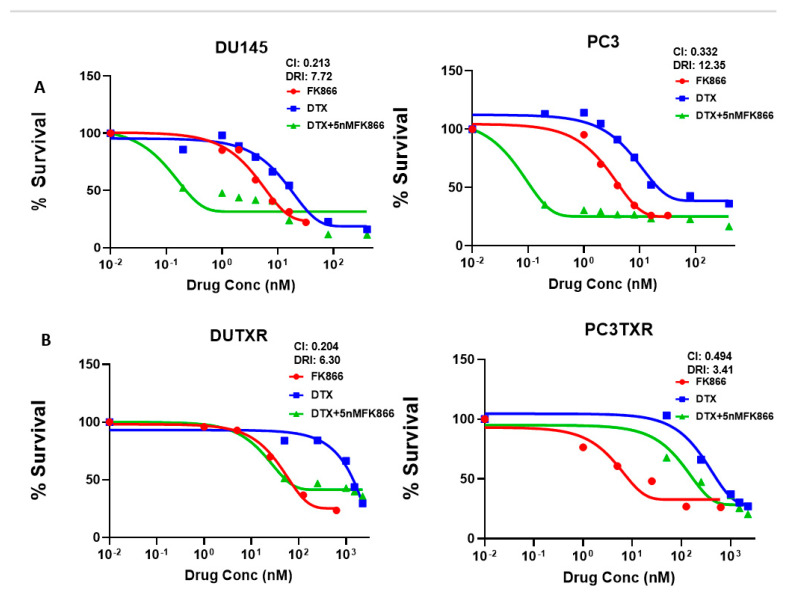

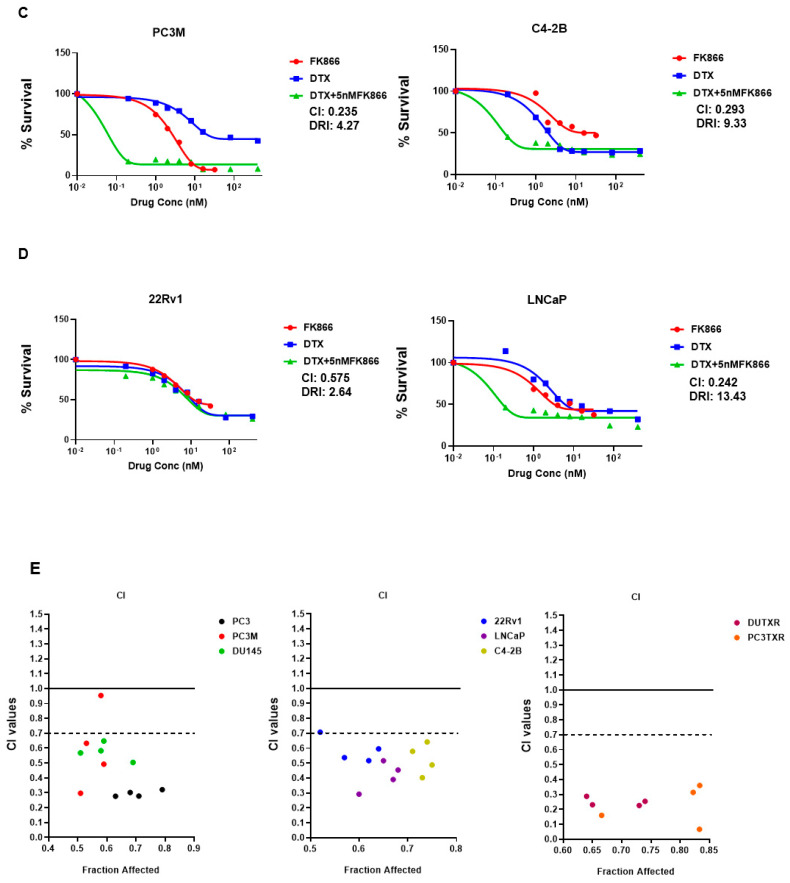

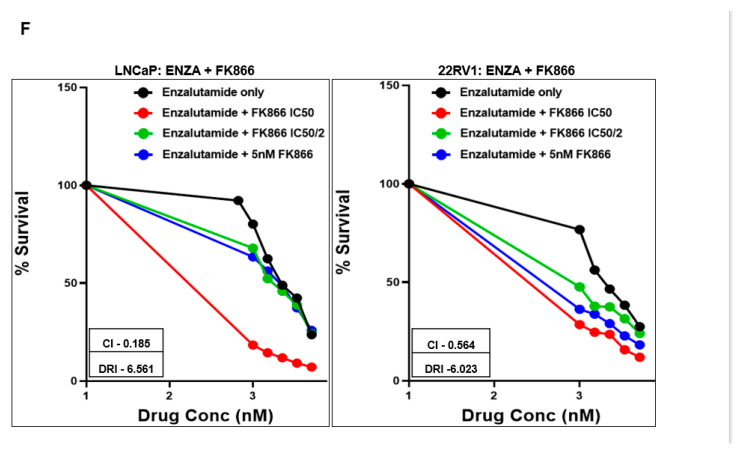
Dose-response curves representing in vitro cytotoxicity of the FK866 drug combination in metastatic PCa cell lines. (**A**–**D**) In vitro cytotoxicity was assessed using MTT or 4,5-Dimethylthiazol-2-yl)-2,5-diphenyltetrazolium bromide assay following treatment with single-agent DTX or single-agent FK866 at increasing concentrations or increasing concentrations of DTX in combination with fixed (5 nM) concentration of FK866. (**A**) AR^lo^ mCRPC cell lines PC-3, DU145, (**B**) Acquired taxane-resistant mCRPC lines DUTXR and PC3-TXR, (**C**) clonally derived metastatic (PC3M), and the AR-independent (C42B) PCa lines; (**D**) AR^high^ mCSPC lines LnCAP and 22Rv1. (**E**) CI values representing synergy between FK866 and TX in metastatic PCa lines (mCRPC lines, mCSPC lines, and Clonally derived mCRPC lines). Combination Index (CI) and Dose reduction (DRI) values were calculated according to Chou-Talalay’s method. CI values between 0.9–0.3 and 0.3–0.1 signify synergism and strong synergism, respectively, between the drugs treated in combination. (**F**) Dose-response plots showing FK866 in combination with the androgen receptor inhibitor Enzalutamide combination in metastatic PCa cell lines.

The effect of DTX, CBZ, and FK866 as single-agent administration on mCSPC, mCRPC cells lines, as well as the acquired taxane-resistant mCRPC line DUTXR cells, were assessed by MTT assay at increasing drug concentrations treatment of each drug. In vitro single-agent cytotoxicity assay results are displayed in Figure S1 as dose vs. %survival. FK866 shows time-dependent decreases in cell survival after 48 h of drug treatment in all the PCa cell lines. Furthermore, IC_50_ values of FK866 were inversely correlated with the IC_50_ values of taxanes, indicating possible synergy (Spearman r < −0.9; *p* = 0.0167). Next, we evaluated the effect of increasing concentrations of DTX + FK866 combination treatment on the AR^lo^ mCRPC cell lines PC-3, DU145, the acquired taxane-resistant mCRPC lines DUTXR and PC3-TXR, the clonally-derived metastatic (PC3M), and the AR-independent (C42B) PCa lines, as well as the AR^high^ mCSPC lines LnCAP and 22Rv1 (Figure 3A–D). The dose-response curves for the drug combinations and CI values indicated high synergy, which was even more profound (CI between 0.2–0.37) in the TX-resistant lines (Figure 3E). Similar highly synergistic results were observed for the FK866 + CBZ combination treatment as well as the acquired taxane-resistant mCRPC (DU145 resistant-DUTXR) cells (Figure S2).

We also demonstrated synergism between FK866 and the AR antagonist Enzalutamide as combination treatment in in vitro models of mCSPC. Figure 3F shows the cell survival curves representing the combination treatment with FK866 and the AR inhibitor Enzalutamide in mCSPC cell lines indicating that FK866 not only synergizes with Taxanes, the combination of FK866 and AR antagonists also showed significant synergy.

### 3.4. FK866 Induced Apoptosis in PCa Cell Lines

**Figure 4:**
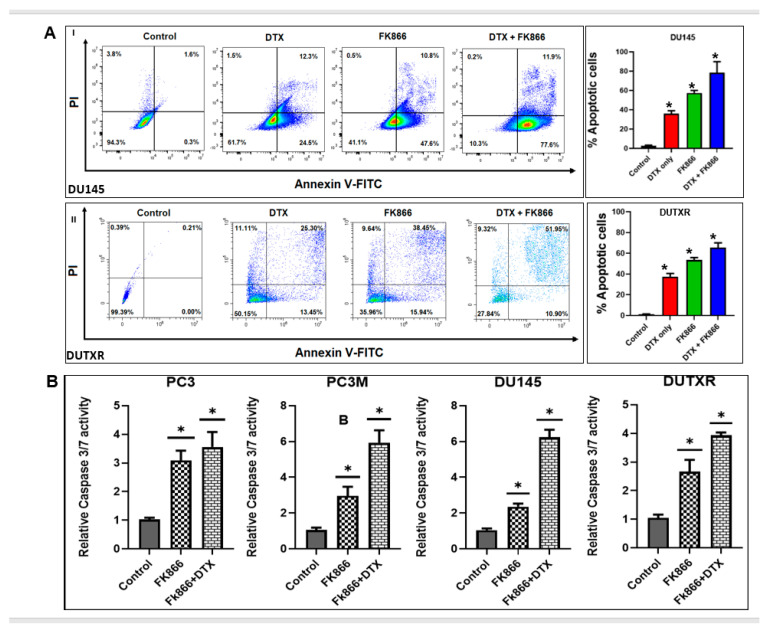

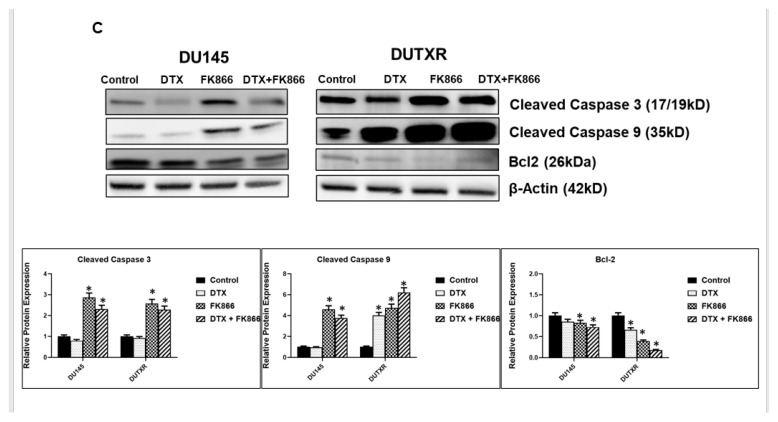
FK866 induces apoptotic cell death in lethal PCa. (**A**) Representative figures showing Annexin-V positive cells measured using flow cytometry following FK866 single-agent and DTX + FK866 combination treatment; bar plot shows higher apoptosis in combination treatment compared to single-agent treatment. (Significance *p*-value * = *p* < 0.05.) Apoptosis was confirmed using (**B**) Caspase-3/7 enzyme activity assay and (**C**) Western blotting and Densitometry analysis showing protein expression of cleaved caspase 9, cleaved caspase 3 and Bcl-2. Representative plots showing DU145 and DUTXR lines are presented. Similar results were obtained for all metastatic PCa cell lines. (Significance *p*-value * = *p* < 0.05). Original uncropped western blot images are provided in Supplementary Figure S8.

To further determine the synergy of these compounds, we performed the assessment of annexin V (a marker of apoptosis) and PI (a marker of necrosis) using flow cytometry in DU145 and DUTXR cells following exposure to FK866 as a single agent as well as in combination with DTX. Our results in Figure 4A showed significant increases in cells staining positive for annexin V and PI following FK866 and FK866 + DTX treatment compared to control and DTX treatment, confirming significantly higher treatment-induced apoptosis post-FK866-treatment. To confirm whether the loss of cell viability following FK866 treatment was indeed due to apoptosis, schedule-dependent effects of FK866 single-agent and combination treatments were determined by measuring the caspase-3/7 activity at estimated IC_50_ (Figure 4B). Our results showed that treatment with FK866 and DTX + FK866 induced apoptosis in every cell line compared to the control (no drug treatment) cells. The relative increase (fold change) in caspase-3/7 activity following were 3.09, 2.96, 2.36, and 2.66 for FK866 single-agent treatment and 3.96, 5.94, 6.23, and 4.66 for FK866 + Taxane combination treatment in PC3, PC3M, DU145, and DUTXR, respectively.

Furthermore, pre- vs. post-FK866 single-agent/combination treatment immunoblotting results of the pro-apoptotic markers cleaved caspase 9 and cleaved caspase 3 corroborated with our caspase 3/7 assay, in addition to the downregulation of the anti-apoptoptic protein Bcl-2 (Figure 4C; original uncropped western blots of Figure 4C are included in the supplementary materials, Figure S8). Interestingly, among all the treatment regimens, the highest level of apoptosis was observed for combination treatment with FK866 + DTX. 

### 3.5. FK866 Treatment Diminished Cell Density and Altered Nuclear Morphology of PCa Cell Lines

**Figure 5:**
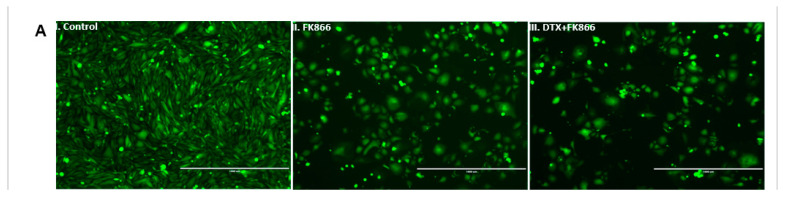

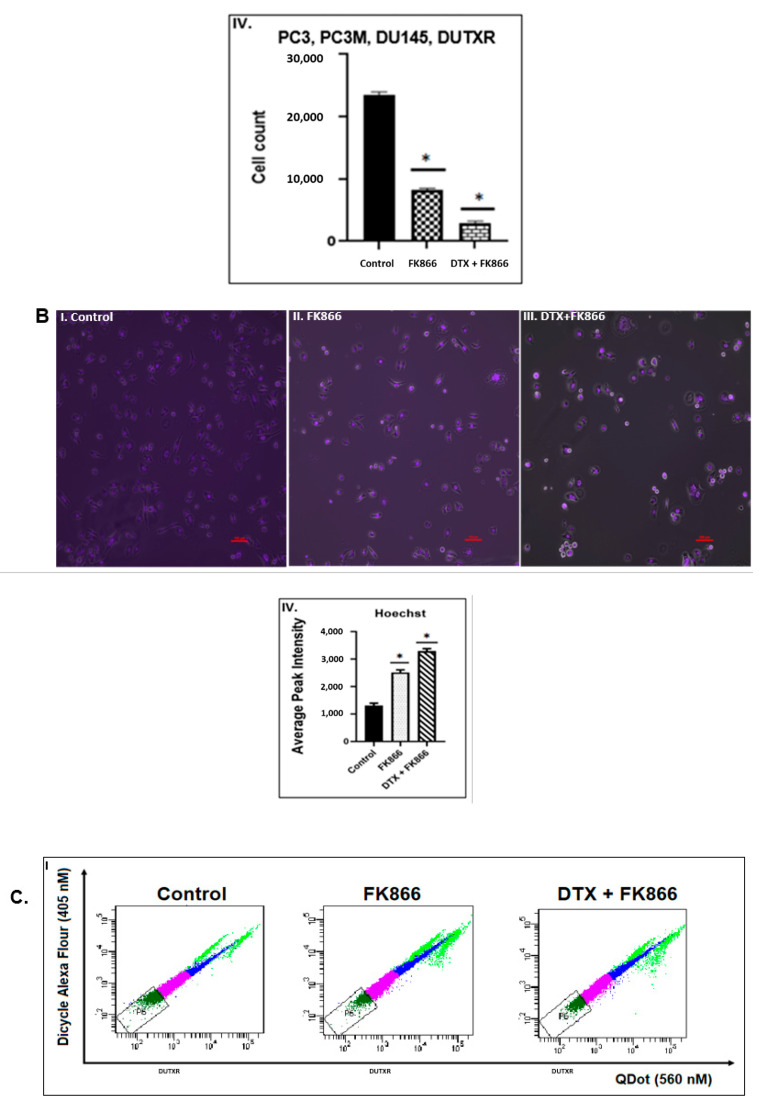

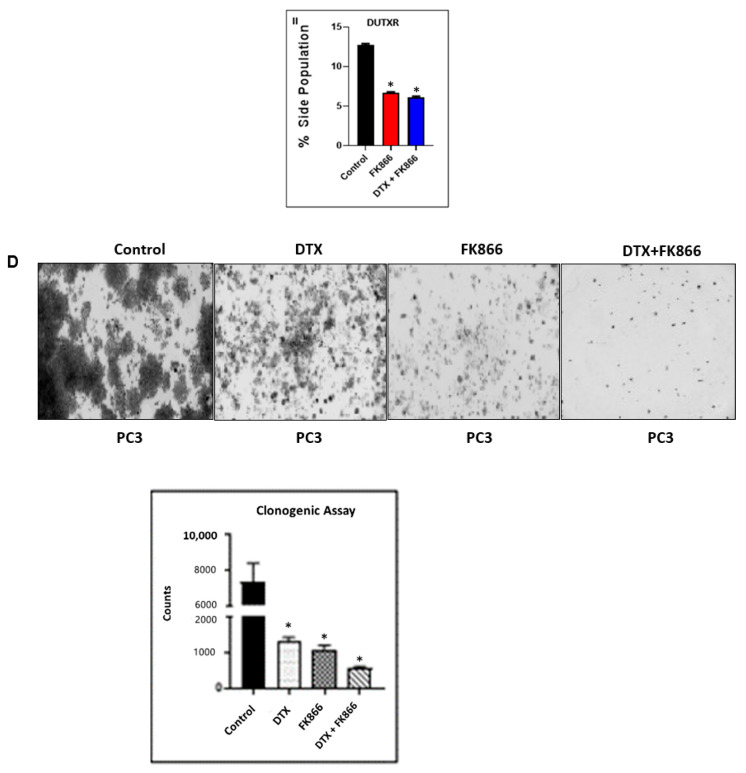
(**A**,**B**). FK866 treatment reduced cell density and changed nuclear morphology. (**A**) Assessment of cellular morphology: I–III. Representative figures showing the effect of the primary (DTX) and secondary (FK866) drugs on cell count and cell morphology of mCRPC cells. The images were captured on the PC3-Luc cells before (0 h) and after (48 h) FK866 treatment either as a single agent or in combination. Microscopy results show significantly higher cell death in combination treatment compared to single-drug treatment for all cell lines; IV. ImageJ data analysis showed a significant difference in cell density for both FK866 single-agent and DTX + FK866 combination treatments. Results show significantly higher cell death in combination treatment at combination dose compared to single-drug treatment for PC3cell lines. (Significant value * = *p* < 0.05.) (**B**) Assessment of nuclear morphology. I–III. Representative figures showing the FK866-based treatment (single-agent and combination with DTX) on cell nucleus morphology of mCRPC cells. NucBlue Live reagent is frequently used to distinguish condensed nuclei in apoptotic cells. The microscopy images were captured on the PC3-Luc cells before (0 h) and after (48 h) following treatment. Microscope images showing treatment effect on the cell lines PC3. Similar results were obtained for all mCRPC lines; IV. ImageJ data analysis showed a significant difference in cell density and nucleus damage for FK866 regimens. (Significance *p*-value * = *p* < 0.05.) Figure 5C,D. FK866 potentially decreased stem cell load in AVPC cells. (**C**) Side population: Representative figure showing side population analysis following FK866 single agent and FK866 + DTX combination treatments in mCRPC reduced load of side population compared to control (no drug treatment). Side populations were gated and selected from the main populations using Vybrant DyeCycle Violet Stain. (**D**) Clonogenic/Colony-forming assay: Representative images in the PC3 cell lines are shown for the control and FK866-treated cells. The treatment was done with primary (DTX) and secondary (FK866) drugs either as a single agent or in combination. Colonies were developed for 21 days.

Finally, we also showed that FK866 treatment reduced mCRPC cell density and changed nuclear morphology. Cells were exposed to FK866 treatment as a single agent as well as in combination with DTX, and cellular morphology was assessed using phase-contrast microscopy. In agreement with MTT assays, micrographs of PCa cells exposed to both FK866 and FK866 + DTX dosing regimens showed decreases in cell density compared to control cells, as shown in Figure 5A. Further, assessment of nuclear morphology of attached cells using NucBlue staining, a reagent frequently used to distinguish condensed nuclei in apoptotic cells, suggested FK866-induced morphological changes like nuclear fragmentation and chromatin condensation, which are indicative of apoptosis (Figure 5B). In addition, our results showed even higher cell death and more nuclear damage in FK866 + DTX combination treatment compared to FK866 single drug treatment. 

### 3.6. FK866 Potentially Decreased Stem Cell Load in Lethal PCa

Side population (SP) cells are a group of enriched progenitor cells thought to have several stem-like characteristics potentially representing PC-CSC phenotype [39]. Therefore, we gated and selected side populations in mCRPC from main populations using Vybrant DyeCycle Violet Stain and assessed the effect of FK866 treatment. Representative figures and summary bar plots in Figure 5C show that FK866 alone or in combination (FK866 + DTX) reduced the side population load in AVPC cell lines.

Next, we evaluated the potential effect of DTX, FK866, and both drugs in combination on the proliferative capacity of the mCRPC/NEPC cells using the colony-forming assay. Cells were treated with FK866, DTX, or combination (FK866 + DTX) for 21 days with supplementation of the drugs every 48 h. Figure 5D shows that while FK866 alone significantly reduced colony number as well as colony size when compared to control or DTX, a combination of FK866 + DTX further reduced the colony numbers.

### 3.7. A Microfluidic Screen Followed by ‘Scratch Assay’ Showed FK866 Is Potentially Effective against EMT Transdifferentiation and Metastasis in Treatment-Refractory Aggressive Subclones 

A Polydimethylsiloxane-PDMS-based μ-channel assay served as a physiologically relevant in vitro metastasis model for screening our top secDrugs. This allowed us to study the effect of our drug combination on tumor cell motility through μ-channels of dimensions that mimic the size of channel-like tracks encountered by migrating cells in vivo [40,41]. Briefly, we fabricated a PDMS-based μ-channel assay using standard multilayer photolithography, and replica molding as previously demonstrated [36,42,43]. The device consisted of an array of parallel channels of variable width (3–50 μm) and with fixed length (200 μm) and height (10 μm). Perpendicular to the μ-channels were two larger 2D-like channels that served as cell seeding and chemoattractant inlet lines (Figure S3). Prior to cell seeding, the μ-fluidic devices were coated with 20 μg/mL rat tail collagen type I (Corning) for 1 h at 37 °C to facilitate cell adhesion. 1–1.5 × 10^5^ vehicle or FK866-treated mCRPC cells were introduced into the cell seeding inlet line via pressure-driven flow and allowed to adhere for 30 min at 37 °C, 5% CO_2_. Next, the cell suspension was removed and substituted with a serum-free medium. The medium, supplemented with 10% FBS, was added into the chemoattractant inlet line to trigger cell entry into the channels. The devices were placed on an automated Nikon Ti2 Inverted Microscope equipped with a Tokai Stage-Top incubator unit, which maintains cells at 37 °C and 5% CO_2_. Cell motility was recorded via time-lapse microscopy. Images were taken every 20 min for 10 h with a 10×/0.45 NA Ph1 objective. To assess the migration efficiency of drug-treated mCRPC cells compared to control, we calculated the percentage of cell entry into the microfluidic channels defined as the total number of cells entering the channels divided by the total number of cells seeded within 50μm diameter from the μ-channel entrances. Because our prostate cancer cells did not frequently enter narrower microchannels (≤10 μm), we focused our analysis on wider channels (≥20 μm).

**Figure 6:**
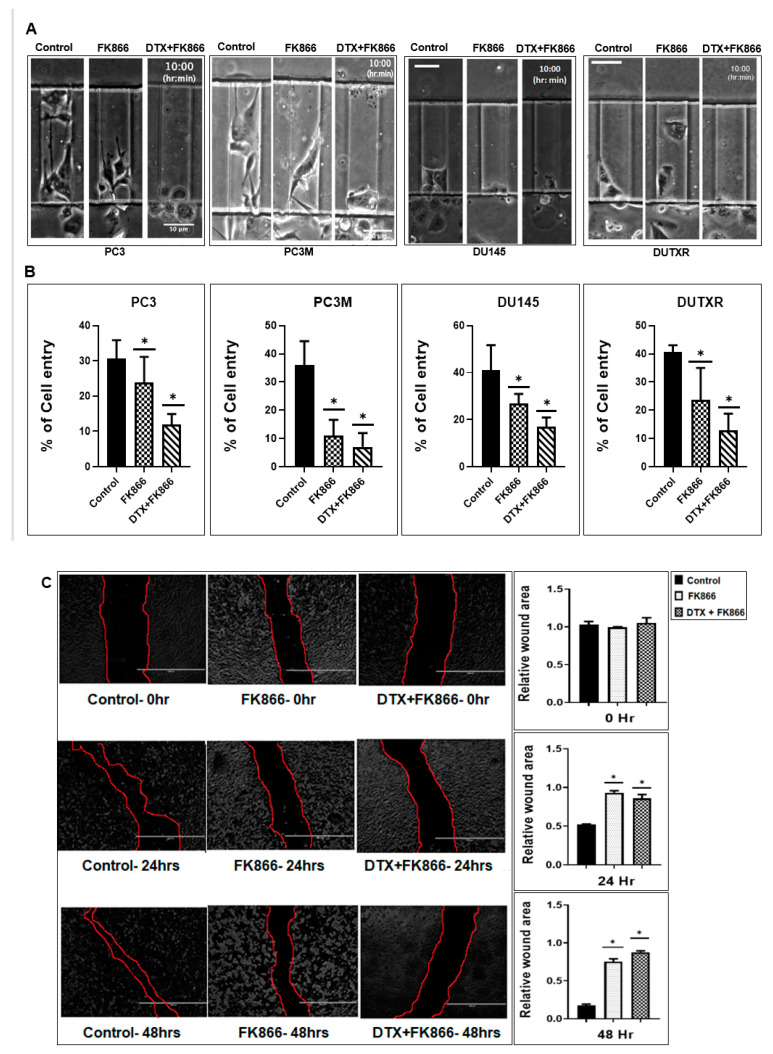
FK866 reduces cell migration and is potentially effective against metastasis and EMT transdifferentiation in lethal PCa. (**A**) Representative images of the control, FK866, and DTX + FK866-treated prostate cancer cells migrating through 50 μm wide μ-channels. The fabrication of a μ-channel assay is described in the Methods section. Reduced cell entry into channels indicates a more generic defect in the migratory capacity of tumor cells. (**B**) FK866 single-agent and combination therapy with DTX reduces the entry of lethal PCa (aggressive mCRPC and acquired taxane-resistant mCRPC lines) into 50 and 20 μm wide μ-channels, indicating a potential role of FK866 in abrogating the metastatic potential. *n* ≥ 3 experiments. (Significance *p*-value * = *p* < 0.05 relative to control.) (**C**) Scratch assay: Representative plots showing results of wound healing (Scratch) assay. Cell migration after 24 and 48 h FK866 single-agent and FK866 + DTX combination were assessed by measuring the scratch size. Images were captured before (0 h) and after (24 h and 48 h) drug treatments (Significance *p*-value * = *p* < 0.05). Bar graphs showed a significant reduction in cell migration (wound healing) following FK866-based single-agent and combination treatments.

Our microfluidic-based cell migration assay revealed that our FK866 single agent and FK866 + DTX combination treatment reduced cell entry into 50 and 20 μm wide channels, suggesting that these interventions may potentially suppress prostate cancer cell invasion and possibly metastasis (Figure 6A and Video S1).

Further, FK866 + DTX combination therapy showed a higher reduction in cell migration in AI-mCRPC/NEPC cells (PC-3, PC-3M, DU145) and acquired taxane-resistant mCRPC (DUTXR) cells compared to single-agent FK866 treatment (Figure 6B). 

To confirm FK866 treatment-induced reduction of mCRPC cell migration, we performed scratch assays by creating a “scratch” in cell monolayer followed by capturing the images at the beginning and regular intervals (0, 24, and 48 h for all treatments—single dose FK866 at the calculated IC_50_ and FK866 + DTX combination at estimated IC_50_) during cell migration to compare the images and quantify the migration rate of the cells to close the scratch. Our results showed that cell migration was higher in control cells compared to FK866 and FK866 + DTX post-treated cells (Figure 6C). Further, combination treatment (FK866 + DTX) had a higher effect in reducing cell migration in PCa cell lines compared to treatment with FK866 alone (*p* < 0.05).

### 3.8. FK866 Showed Selective on-Target Inhibition of NAMPT Activity and a Distinct Impact on Gene Expression Signature 

**Figure 7:**
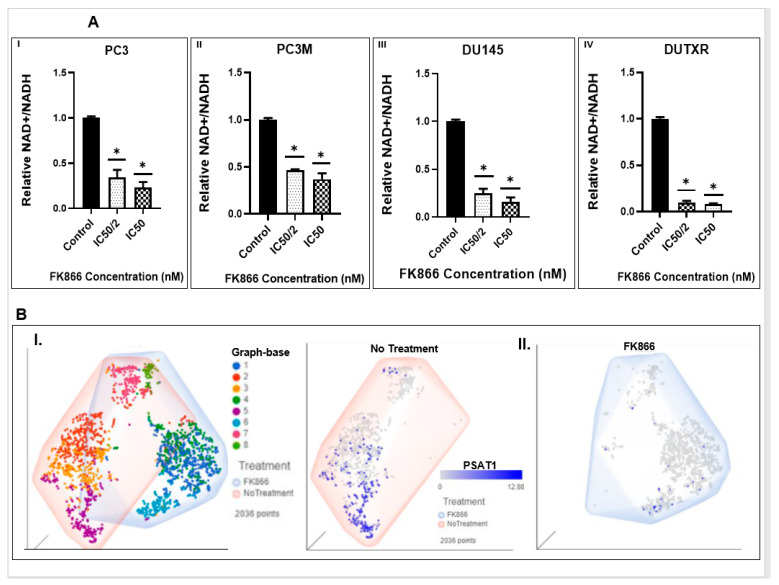
FK866 showed selective on-target inhibition of NAMPT activity. (**A**) FK866 post-treatment NADH activity: Effect of FK866 on total cellular NAD^+^/NADH ratio in PCa cell lines was detected by NADH activity assay in AR^lo^ mCRPC (I. PC3, II. PC3M, III. DU145) and in Acquired taxane resistant mCRPC (IV. DUTXR) cell lines treated with FK866 doses equivalent to IC50 and IC50/2 of each cell lines. A significant decrease in the NAD^+^/NADH ratio was observed compared to control cells after 24 h of treatment. Results are presented as mean ± SD; *n* = 3 for each experiment (* *p* < 0.05, compared to untreated controls). (**B**) Pre- vs. Post-treatment scRNAseq data analysis: Effect of NAMPT inhibitor (FK866) on the sub-clonal population of human prostate cells in vitro following single-cell transcriptomics analysis. I. Pre- vs. post-treatment t-SNE clusters, showing II. Erosion of the PSAT1^high^ cluster (Cluster 5) in the FK866-treated scRNAseq data.

NAD/NADH activity assay: The effect of FK866 on its intended target, NAMPT, was assessed using NAD/NADH-Glo activity assay that measures the ratio between total oxidized and reduced nicotinamide adenine dinucleotides (NAD^+^ and NADH, respectively). We found that FK866 selectively inhibited the total cellular NAD^+^/NADH ratio in all PCa cell lines in a dose-dependent manner. Figure 7A demonstrates a significant (*p* < 0.05) decrease in NAD^+^/NADH ratio following 24 h FK866 IC_50_ and IC_50_/2-treatment compared to the control cells. (Range: 2.70 to 12.35 for IC_50_, and 2.15 to 10.01 for IC_50_/2.) 

Pre- vs. post-FK866-treatment scRNAseq analysis: The on-target effect of FK866 was confirmed at the single-cell level using post-FK866-treatment scRNAseq datasets compared to the baseline/untreated PCa (Figure 7B). Our single-cell transcriptomic analysis showed that FK866 treatment resulted in the loss of the tSNE cluster 5 (the PSAT1^high^ cluster) following FK866 treatment represented by high expression of PSAT1 gene, indicating FK866-induced downregulation of PSAT1 gene, which is a downstream protein in NAMPT-mediated NAD salvage biosynthesis pathway.

**Figure 8:**
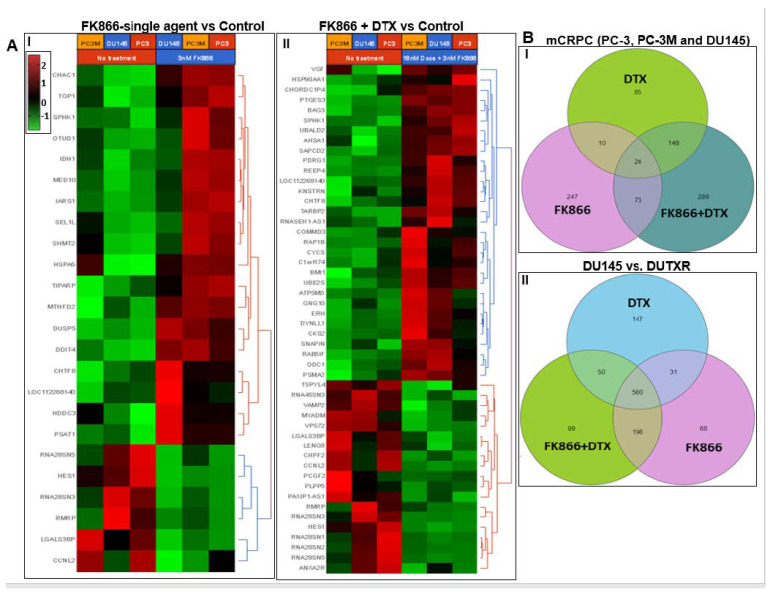

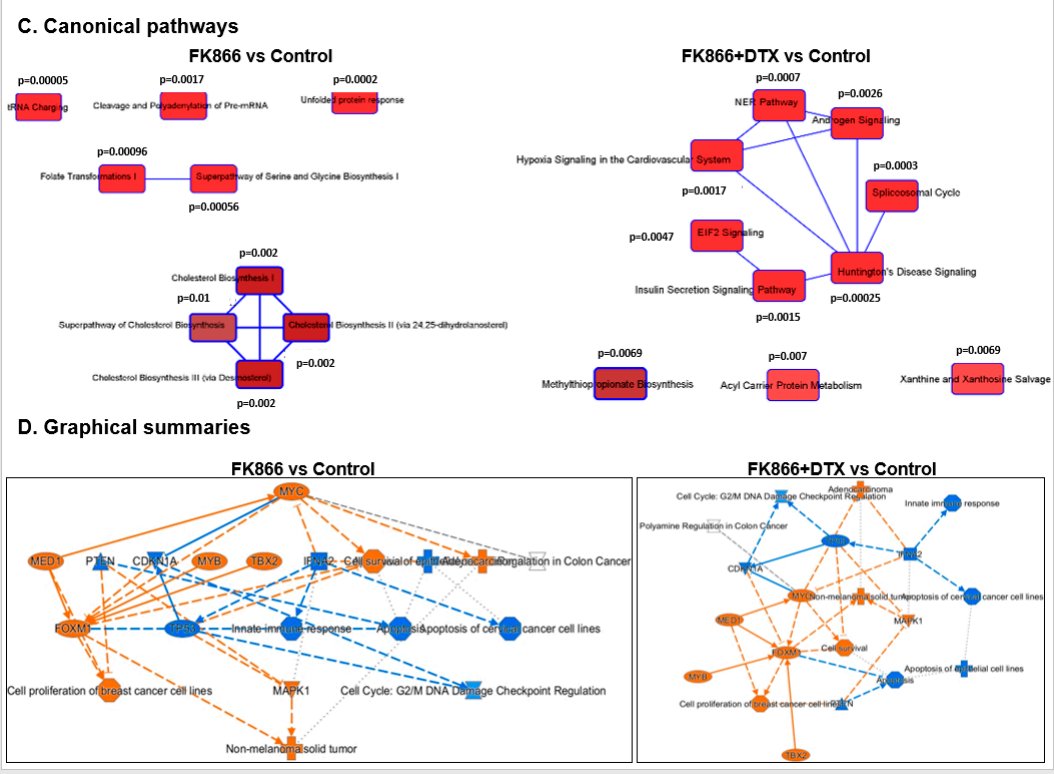
Differential gene expression profiling analysis results. (**A**) Heatmaps representing top differentially expressed genes (DEGs) following DTX, FK866 single-agent, or DTX + FK866 combination treatments in human mCRPC cell lines (*n* = 3), 24 h following drug exposure. (I) FK866 single-agent treatment. (II) DTX + FK866 combination treatment. Log_2_ ratios are depicted in a color scale where red represents upregulation and green represents downregulation. Columns represent cell lines and rows represent genes. Prior to hierarchical clustering, gene expression values were filtered (samples with max TPM < 1 were removed) and z-score normalized. (**B**) Venn diagrams representing unique and common DEGs (*p* < 0.05) between DTX, FK866, and DTX + FK866 treatments among: I. All mCRPC lines; II. DU145 vs. DUTXR lines in all cell lines. Ingenuity pathway analysis: IPA analysis was performed based on the top DEGs following FK866 single-agent of FK866 + DTX combination treatments. (**C**) Top canonical pathways. (**D**) Graphical Summaries.

Pre- vs. post-FK866-treatment bulk RNAseq analysis: Next, we performed global whole-transcriptome profiling by bulk tumor RNAseq to compare changes in gene expression induced by FK866 in AVPC cell lines. GEP data were normalized to the baseline (no-treatment). Volcano plots in Figure S4 show differentially expressed genes (DEGs) following DTX, FK866 single-agent or combination treatment in human mCRPC cell lines. Heatmaps were generated following differential gene expression analysis (Figure 8A). A total of 247 genes were uniquely differentially expressed above the significance threshold (*p* < 0.05) at 48 h post-FK866 single-agent treatment, while 85 and 289 genes were differentially expressed following DTX single-agent and FK866 + DTX combination treatments, respectively (Figure 8B). 

Table S2 lists the top differentially expressed genes (DEGs). The top 10 downregulated DEGs between pre- (untreated; 0 h) vs. 48 h post-treatment, irrespective of treatment type (FK866, FK866 + DTX, or DTX), were C1S, IFITM3, FAM229A, LHFPL5, DUS4L-BCAP29, ZACN, LGALS3BP, CXCL2, HES1, and FAS. On the other hand, the top upregulated DEGs were ELMOD1, SESN3, CYP1A1, DDIT4, SLC7A11, FTCDNL1, LAMP3, BHLHA15, PAPPA2, and EML5. 

Among these, HES1 was downregulated in all treatment groups, with the highest level of downregulation (~6-fold) following combination treatment (FK866 + DTX). FAM229A, LGALS3BP, LY6G5B, MAT2A, LTB4R, NSUN5P2 were downregulated in both FK866 single-agent and combination (FK866 + DTX) groups, while PITPNC1 was upregulated in both the groups, albeit at different levels. 

IPA analysis performed based on the top DEGs associated with FK866 single-agent treatment revealed Inhibition of Matrix Metalloproteases (*p* = 0.0059), oxidative stress, and cell cycle among the top dysregulated pathways (Figure 8C). Further, ATF4 (*p* = 0.000000000397) was predicted as the top upstream regulator. Interestingly, G2/M DNA Damage Checkpoint Regulation (*p* = 0.0086) and Kinetochore Metaphase Signaling Pathway (*p* = 0.005) were among the top canonical pathways inferred from the following genes differentially regulated between FK866 + DTX vs. baseline: MED1, FOXM1, MAPK1, MYB, TBX2, CDKN1A, PTEN, TP53, TFNA2. Further, IPA predicted IFNA2 as the top upstream regulator based on the expression of genes (Figure S5A). Among microRNAs, IPA predicted mir-1-3p as the top upstream regulator based on the expression of target genes and IFNA2 (Figure S5B). 

The graphical summaries in Figure 8D show that FK866-based treatment regimens induce upregulation of apoptosis in tumor cell lines, as well as the genes like STAT1, IRF1, DDX58, TNF, IFNG, and downregulation of tumorigenesis in tissues, polyamine regulation, and the genes FOXM1, MAPK1, AREG, etc. A detailed heatmap comparing differentially regulated pathways between FK866 single-agent and combination treatments are provided in Figure S6, including differential regulation of the following pathways between the treatment regimes Putrescine Biosynthesis, Prostanoid Biosynthesis, Eicosanoid Signaling, Cell cycle-G2/M damage, Kinetochore Metaphase signaling, Polyamine regulation, Phosphatidylethanolamine Complement system, P53 signaling, tRNA charging, Matrix metalloprotein, and Ferroptosis Signaling Pathway.

### 3.9. FK866 Treatment Results in Differential Regulation of Cell Cycle and Checkpoint Regulation Genes 

**Figure 9:**
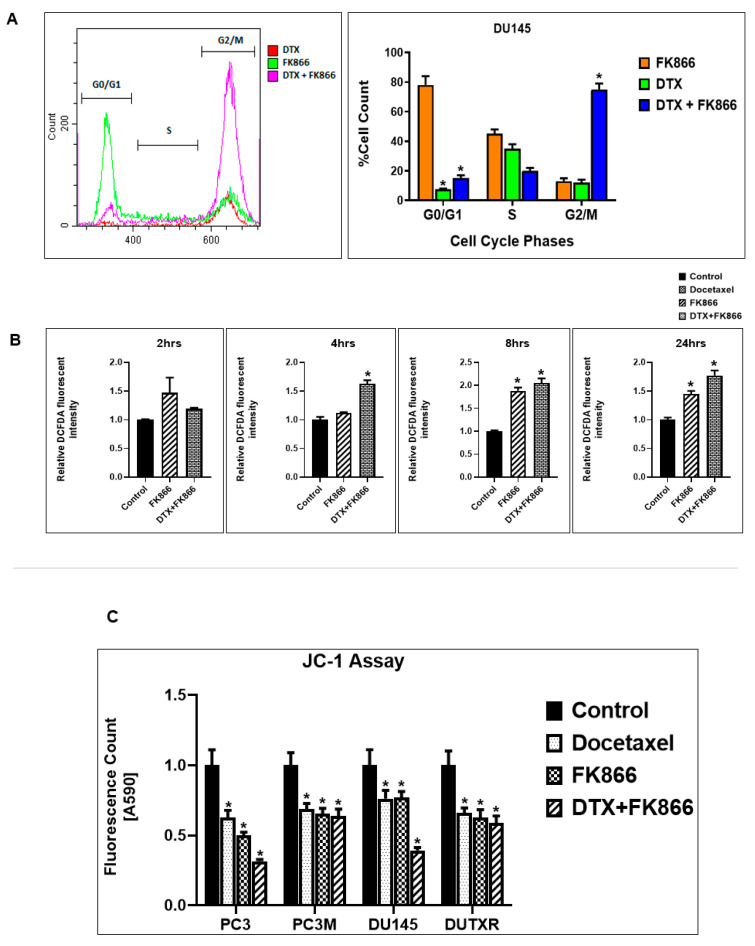

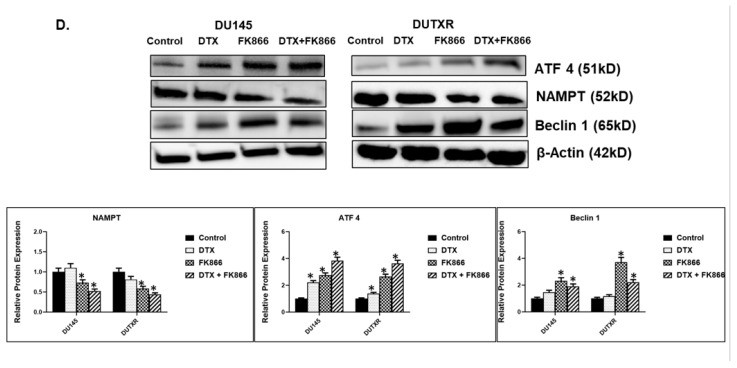
Functional validation of top key pathways. (**A**) Effect of FK866 treatments on Cell cycle: Representative figure showing results on the DU145 cell line. Cells were exposed to DTX, FK866, and DTX + FK866 treatment for 24 h, stained with propidium iodide (PI), and cell cycle phases were assessed by flow cytometry. Cell cycle stages were analyzed by CytExpert (Beckman Coulter). FK866 was shown to arrest cell cycle at the G2/M checkpoint. Cell Cycle in treatment groups was normalized to the corresponding control. (Significance *p*-value * = *p* < 0.05). Data are presented as the mean ± SEM of three separate experiments (*n* = 5/study). (**B**) FK866 post-treatment ROS generation measured by DCFDA assay: ROS generation was measured after 2, 4, 8, and 24 h DTX, FK866 single-agent, and in DTX + FK866 combination treatments. (**C**) Mitochondrial Membrane Potential: FK866 treatment-induced mitochondrial dysfunction was measured by JC-1 Assay. (**D**) Western blotting and Densitometry analysis: Protein levels of FK866 target NAMPT, and ER stress and Autophagy markers Beclin-1 and ATF-4 were measured after DTX, FK866, and DTX + FK866 treatment. Post-treatment FK866 exhibited downregulation of NAMPT, and upregulation of Beclin-1 and ATF-4 compared to control (no drug treatment). (Significance *p*-value * = *p* < 0.05.) Original uncropped western blot images are provided in Supplementary Figure S9.

Since cell cycle and checkpoint regulation were significant among the IPA-predicted signaling pathways, we performed in vitro assessment of cell population in each cell cycle checkpoint (G2/M) following FK866 single-agent and DTX combination treatment using Flow cytometry. This effect of FK866, either alone or in combination, on the cell cycle distribution was assessed by quantifying DNA content. We observed that treatment of PCa cell lines with FK866 resulted in G2/M checkpoint arrest. Furthermore, a higher number of cells were arrested at G2/M following combination treatments compared to FK866 or DTX alone (Figure 9A). Treatment of cells with FK866 + DTX increased the highest percentage of cells in the M phase (*p* < 0.05) with a concomitant decrease in S and G0/G1 populations.

### 3.10. FK866 Treatment Promotes Oxidative Stress and Mitochondrial-Mediated Pathway Gene Dysregulation

Among the IPA-predicted pathways following FK866 treatment were oxidative stress and mitochondrial dysfunction. Intracellular ROS levels are the indicator of oxidative stress. Therefore, to validate the mechanism of action of the drug combination, we quantified the intracellular ROS level in the pre-and post-treatment condition of FK866 as a single-agent vs. combination with DTX using the fluorogenic probe 2,7-dichlorofluorescein diacetate (DCFDA), a cell-permeable non-fluorescent probe that shows fluorescence when it is oxidized. Cellular superoxide anions were measured by using the fluorescent dye DHE. Figure 9B depicts significant ROS generation following 2, 4, 8, and 24 h DTX or FK866 single agent and DTX + FK866 combination treatments. Further, combination treatment exhibited higher ROS generation than single-agent treatment and control in Acquired taxane-resistant AI-mCRPC (DUTXR) cell lines (*p* < 0.05). Mitochondrial membrane potential and FK866 treatment-related mitochondrial dysfunction were accessed using JC-1 (Sigma). JC-1 is a cationic carbocyanine dye that accumulates in mitochondria. FK866 treatment enhanced mitochondrial dysfunction in AR^lo^ mCRPC/NEPC (PC-3, PC-3M, DU145) and acquired taxane-resistant AI-mCRPC (DUTXR) cell lines (Figure 9C). 

To confirm the results of our differential gene expression analysis, IPA pathway analysis, and in silico patient data validation analyses, we selected ATF4, Beclin 1, and NAMPT for immunoblotting in PCa cell lines. Figure 9D shows immunoblotting results confirming upregulation of ATF4 and Beclin 1 and downregulation of the FK866 target gene NAMPT following FK866-based treatment (original uncropped western blots of Figure 9D are included in the Supplementary Materials Figure S9). 

### 3.11. Validation of FK866 Treatment-Induced Gene Signatures Using Patient Datasets

**Figure 10:**
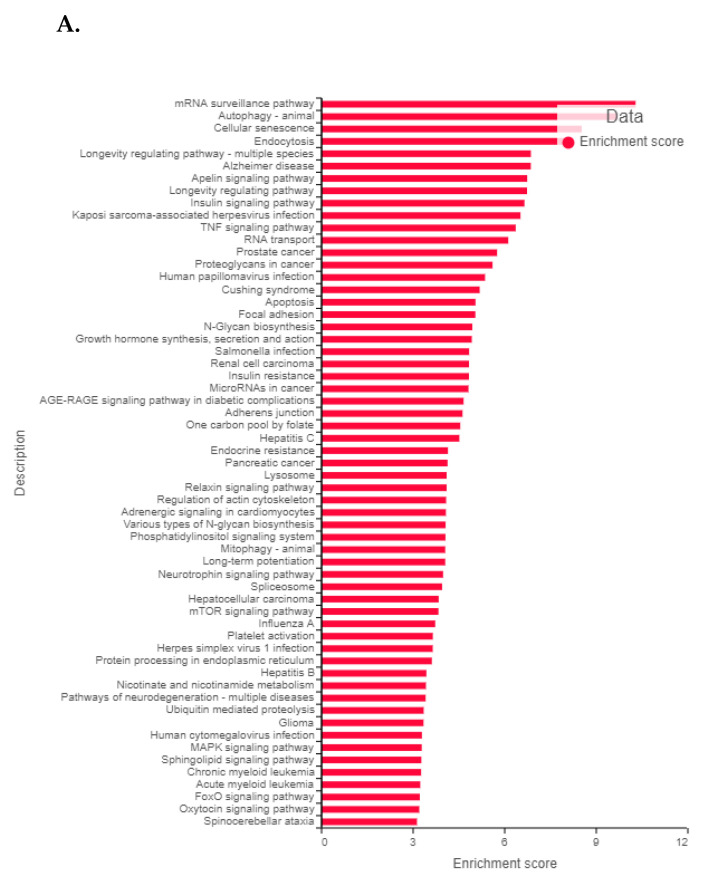

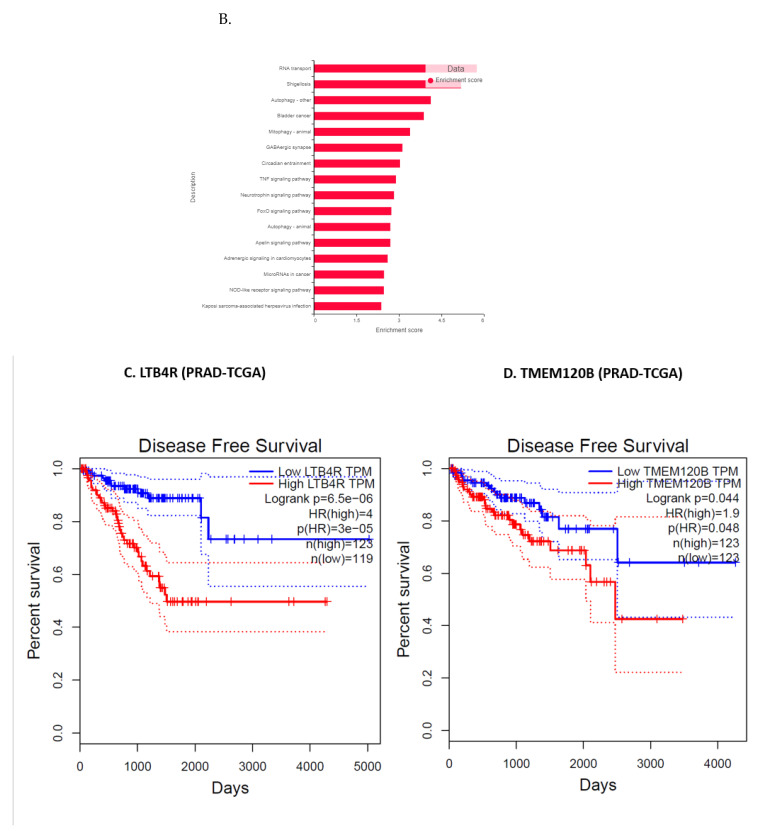
Validation of FK866 treatment-related gene signatures using patient cohort datasets. Reverse-matching using patient cohort datasets show FK866 treatment has the potential to reverse PCa lethality. Pathway analysis was performed based on the top DEGs in (**A**) PCa patient cohort; (**B**) Top FK866 treatment-induced upregulated pathways that were significantly downregulated in PCa patients with BCR. Validation using TCGA’s prostate adenocarcinoma (PRAD) GEP dataset: In silico analysis of the top FK866 treatment-induced downregulated pathways (Table S3) that were significantly downregulated in PCa patients with BCR using TCGA’s PCa dataset: Kaplan-Meier Curves showed that the genes (**C**) LTB4R, and (**D**) TMEM120B were significantly associated with disease-free survival.

RNAseq data on PCa patients were obtained from the Gene expression omnibus database (GSE54460) [31]. The dataset includes 100 PCa patients (49 with BCR, 51 with no BCR) from the Atlanta VA Medical Center, Moffitt Cancer Center, and Sunnybrook Health Science Center. A summary of the clinically relevant parameters in the PCa patient dataset that apply to the clinical samples, including Gleason score, BCR, pSpTage, age and race is provided in Table S3. First, we performed differential gene expression analysis between patients with or without biochemical recurrence (BCR). Figure 10A shows the top pathways that were significantly different between BCR vs. no-BCR based on DEGs with *p* < 0.05. Next, as a reverse-matching approach, we compared the list of shared dysregulated (down or upregulated) genes with our list of top FK866-treatment-induced DEGs. Table S4 lists the genes that were up-or down-regulated in PCa patients with BCR AND had significant fold changes in the opposite direction following FK866 treatment in our model systems, indicating that FK866 might be capable of reversing the input signature in the patient cohort. The top genes that were significantly upregulated in patients with BCR and showed significant downregulation following FK866 treatment in PCa cell lines were LTB4R, IFITM3, and TMEM120B. Furthermore, C1S (3.47-folds; *p* = 0.004), in addition to LT4BR (2.23-fold; *p* = 0.04), were significantly upregulated in patients with high Gleason score (8, 9 or 10) and among the top downregulated genes following FK866 treatment in cell lines. Finally, Figure 10B shows that several FK866 treatment-induced pathways were significantly downregulated in PCa patients with BCR.

Interestingly, additional in silico analysis using TCGA’s prostate adenocarcinoma (PRAD) GEP dataset showed that the genes LTB4R and TMEM120B were also significantly associated with disease-free survival (Kaplan-Meier curves are presented in Figure 10C,D), with Hazards Ratios 4 (*p* = 0.00003) and 1.9 (*p* = 0.048), respectively. Thus, this biomarker-based method served as a novel tool to screen drugs against aggressive PCa.

## 4. Discussion

Drug development for aggressive and/or lethal treatment-resistant PCa poses a significant challenge with very few therapeutic successes [11,22]. In this study, we introduced a pipeline that integrated a pharmacogenomics data-driven approach with scRNAseq-based rapid drug screening method and identified FK866 as a proof-of-concept secondary drug (‘secDrugs’) against lethal PCa including aggressive, acquired taxane resistant, and stem-like cell types representing NEPC and stem-like (EMT) phenotypes. Notably, we used scRNAseq as an innovative approach to demonstrate that a subset of AR^low^ PCa cells in metastatic prostate cancer, including castration-sensitive and castration-resistant tumors, harbored signatures of Epithelial-mesenchymal transition (EMT) and cancer ‘stemness’ which we also showed as targets of FK866. Thus, using a ‘Double-Hit method’ that integrates single-cell transcriptomics (scRNAseq) and pharmacogenomics-guided computational prediction (secDrug), we predicted that FK866 (NAMPT inhibitor) targets the majority of the PCa single cells representing cancer stemness, drug resistance, aggressiveness/CRPC, AR receptor or neuroendocrine status, and is, therefore, a top drug candidate potentially effective against most lethal forms of PCa. 

Further, using cell-based assays on in vitro models of PCa and clinical data, we demonstrated that FK866 has the potential to be repurposed as novel candidate drugs for treating lethal PCa, particularly in PCa models of Taxane and/or AR-inhibitor drug resistance and cancer stemness.

Earlier studies have reported that FK866 is a NAMPT inhibitor and controls cancer progression by a downregulation of TNFα, IL-6 expressions, CXCR4 [44]. Tumor cells have increased requirements for NAD^+^, an essential cofactor in cellular processes, including cellular metabolism (oxidative phosphorylation), protein modification, and messenger synthesis [45]. Therefore, NAMPT inhibitors play a key role in energy metabolism in cancer by specifically inhibiting the biosynthesis of NAD^+^ from niacinamide [28,29]. 

Furthermore, recent insights have revealed several additional targetable cellular processes that are impacted by inhibition of NAMPT, such as—sirtuin function (tumor cell proliferation and progression), DNA repair machinery, redox homeostasis (ROS), and immune processes [44,46,47,48,49]. High NAD^+^ and NAMPT expression have also been shown to be associated with the presence of a higher proportion of cancer-initiating/stem cells. Consequently, NAMPT inhibition has been shown to result in the loss of cancer stem cells through excess autophagy resulting in the disruption of the maintenance of cancer-cell stemness [50,51]. Daporinad (FK866/APO866) is currently under clinical trials in several cancers. In fact, previous literature on FK866 and PCa also supports the potential effectiveness of FK866 in prostate cancer, including inhibition of the growth of PCa-engineered tumors by interference with energy metabolism [28,29]. FK866 was considered safe and well tolerated and showed potential anti-angiogenic properties and dose-dependent decrease of patient VEGF levels in a Phase I clinical study that treated 24 patients with advanced Cutaneous T-cell Lymphoma (NCT00431912).

Since FK866 is an inhibitor of NAD biosynthesis, we confirmed that FK866 selectively inhibited NAD^+^/NADH ratio in PCa cell lines in a dose-dependent manner. Interestingly, we observed >2-fold high baseline NAMPT mRNA expression in castration-resistant lines compared to castration-sensitive lines. This higher baseline NAMPT activity (and consequently NAD/NADH ratio) in mCRPC lines may be related to the comparatively lower FK866 single-agent cytotoxicity in the castration-resistant lines.

Further, we combined a novel micro-fluidic-based cell migration assay, genome-wide bulk inter-tumor (RNAseq), and single-cell transcriptomics (scRNAseq) analysis to elucidate in detail the treatment-induced genes and molecular pathways/networks underlying the FK866 mechanism of action and its potential impact on tumor metastasis, migration, invasion, intracellular ROS activity, autophagy and most importantly, ‘cancer stemness’, in AVPC. 

Among the top downregulated genes were the potential proto-oncogene IFITM3 (<−7-fold), C1S (<−8-fold), FAS (<−3.6-fold), as well as LGALS3BP, LTB4R, MAT2A, and CXCL2.

Interferon-inducible Transmembrane Protein 3 (IFITM3) has an oncogenic role and promotes cell proliferation, cell migration through EMT, invasion stemness, bone metastasis, and PCa progression through activation of a novel TGF-β-Smads-MAPK pathway [52,53]. Therefore, IFITM3 expression is correlated with poor prognosis of PCa, as well as tumorigenesis, progression, differentiation, and tumor relapse [54,55]. Concurrently, we showed that baseline IFITM3 gene expression was upregulated in PCa patients with biochemical recurrence compared to patients without any BCR event. Furthermore, IFITM3 expression was also found to be significantly higher in TCGA-PRAD patients (>1000 folds; *p* = 0.00992) with African ancestry compared to Caucasians. Since African American (AA) men are disproportionally affected, more likely to develop PCa, less likely to respond to conventional therapies, and twice as likely to die from PCa compared to other ethnicities, this indicates a possible role of high IFITM3 expression in these inter-ethnic differences [2,56]. In our study, we observed ~7-fold downregulation of IFITM3 gene expression following FK866 treatment.

High expression LTB4R has been reported in various cancers, including PCa [57]. Interestingly, we also observed that higher expression of LTB4R is associated with poorer disease-free survival (DFS) in the TCGA-PRAD PCa patient cohort. Our observed downregulation of LT4BR following FK866-based treatment regimens indicates its potential efficacy in improving patient survival. Thus, through reverse-matching, we showed that FK866 has the potential to reverse the effects of genes/pathways that were significantly dysregulated in PCa patients with a lethal/aggressive form of the disease. 

Gene expression level of the complement protein C1S is significantly elevated in cancers and promoted cancer cell proliferation along with poor prognosis [58]. FAS protein overexpression indicates poor biochemical recurrence (BCR)-free survival in PCa [59,60]. Additionally, the androgen receptor directly binds to the Fas/FasL domain and promotes the androgen-independent growth of PCa [61]. LGALS3BP has earlier been shown to be upregulated in human colorectal and prostate cancer which may influence oncogenesis and promote cancer growth and angiogenesis through the PI3K/AKT/VEGFA pathway [62]. MAT2A plays a crucial role in various cancer progression, including PCa [63,64]. Inhibition of MAT2A gene expression repressed the growth of human PCa [65]. 

Further, our study also showed that FK866 downregulates CXCL2 expression (Fold change < −3.5). The CXCL8 axis is beneficial for the regulation of cancer cell progression [66]. We also report that FK866 downregulated FOS, ATP1B3, and GOLGA8B, which have known benefits in PCa treatment. C-Fos is upregulated in advance PCa and correlated with Erk MAPK pathway activation with disease recurrence [67,68,69]. ATP1B3 expression was increased in various cancers, including PCa, and increased cell proliferation, migration, apoptosis, and epithelial-to-mesenchymal transition (EMT) of cells [70,71]. Recent studies reported that high expression of (GOLGA8B) is associated with PCa progression and poor prognosis [72].

Although the number of FK866 treatment-induced upregulated genes were lower, the list of top genes included GABARAPL1, OTUD1, CHAC1, and SESN3. A recent study reported that elevated levels of GABARAPL1 suppress metastasis and cell proliferation through PI3K/Akt pathway in PCa [73,74]. High expression levels of OTUD1 were associated with improved prognosis in non-small cell lung cancer and adenocarcinoma [75]. While CHAC1 inhibits cell viability and increases the sensitivity to DTX for PCa [76] and is associated with autophagy marker ATF4 [77,78]. We observed treatment-related downregulation of CHAC1 as well as ATF4. Further, overexpression of SESN3 was associated with cancer-cell proliferation suppression through mTORC1, Oxidative Stress, and Autophagy pathway [79]. Additionally, we also observed that FK866 treatment upregulated MERTK, APLF, which is beneficial for treatment response against mCRPC. A recent study reported that MERTK regulates PCa dormancy, which inhibits PCa growth and increases metastasis-free survival [80]. Further, APLF suppression promotes breast cancer and bladder cancer invasiveness through inhibition of proliferative capacity, altered cell cycle behavior, induced apoptosis, and impaired DNA repair ability [81,82].

Traditional gene fusions are involved in the development of various cancer. DUS4L-BCAP29, a chimeric fusion RNA, has been reported to be a cancer-fusion in prostate and gastric cancer, which play a tumorigenic role [83,84,85]. We observed >4.5-fold downregulation of DUS4L-BCAP29 following FK866 treatment. Finally, our pathway analysis predicted mir-1-3p as the top upstream regulator based on DEGs. Interestingly, several recent studies have reported that miR-1-3p is a tumor suppressor and regulates PCa aggressiveness [86,87,88,89]. A recent study demonstrated that enhanced expression of Androgen Receptor transcript variant 3, also known as AR-V7 (ENST00000504326 or NM001348061), is associated with resistance to 2nd-generation AR signaling inhibitors [90]. We performed transcript count comparison and observed 1.5- to 6.3-fold downregulation of AR-V7 following FK866 treatment compared to untreated PCa cells with detectable AR-V7 levels.

Importantly, we identified HES1 as one of the top genes downregulated following FK866 treatment. HES1 gene encodes Transcription factor HES1 (hairy and enhancer of split-1) protein. HES1 plays a critical role development of cancer stem cells (CSCs), cancer metastasis, and multidrug resistance in many cancers [91]. Overexpression of HES1 PCa has been shown to play a crucial role in PCa progression [92,93,94]. 

Cancer metastasis is characterized by the dissociation of cancer cells from the primary tumor site and colonization in a distant organ by traveling through interstitial tissues, intravasation into the blood or lymphatic vessels, and extravasation into a new site followed by proliferation and plasticity, a common feature of EMT [95,96]. Thus, cell motility through confining pores plays a pivotal role in the process of metastatic dissemination, during which cells undergo EMT [97,98,99]. Our innovative microfluidic-based approach using a physiologically relevant model of in vitro metastasis showed a significant decrease in migration efficiency of FK866-treated lethal PCa cells compared to control, confirming a potential role of FK866 in cell migration and tumor metastasis. Furthermore, treatment with FK866 also impacted JAK-STAT signaling pathway (Supplementary Figure S7) in addition to the EMT markers described above, which is in agreement with a recent study investigating the impact of new treatments on EMT markers and cancer stemness [100].

## 5. Conclusions

Using an innovative approach that integrates single-cell -omics technologies, microfluidics, and tumor mRNA sequencing with In vitro studies and patient data-based validation, we conclude that FK866 has the potential to improve the clinical outcome in AVPC chemotherapy by enhancing the therapeutic efficacy and abrogating the possibilities of development of bulk and subclonal drug resistance. Such an evidence-based approach promises to minimize the chances of trial failures and improve the probability of clinical success.

Since our approach is primarily focused on using in vitro model systems, further preclinical validation and single-cell multi-omics strategies using mouse xenograft models and patient-derived organoids are warranted to build upon our findings. Further, this will also allow the understanding of subclonal molecular pathways underlying differential patterns of PCa aggressiveness and drug response between AA vs. CA men.

Overall, our study creates a pipeline to introduce secDrugs as potent clinical-trial-ready therapeutic options for the management of lethal PCa with stem-like features.

## Figures and Tables

**Table 1:** Top drugs (‘secDrugs’) derived from our pharmacogenomics data-driven analysis.

	Drug Name	Target	Target Pathway
1	**Afatinib**	ERBB2, EGFR	EGFR signaling
2	**AKT inhibitor VIII**	AKT1, AKT2, AKT3	PI3K/AKT pathway
3	**AMG-706 (Motesanib)**	VEGFR, RET, KIT, PDGFR	RTK signaling
4	**AZD6482**	PI3Kβ	PI3K/MTOR signaling
5	**Cetuximab**	EGFR	EGFR signaling
6	**CP724714**	ERBB2	RTK signaling
7	**FH535**	PPARγ, PPARδ	Wnt/β-catenin signaling
8	**FK866**	NAMPT	NAD^+^ salvage pathway
9	**GSK2126458 (Omipalisib)**	PI3K (class 1), MTORC1, MTORC2	PI3K/MTOR signaling
10	**GW441756**	NTRK1	RTK signaling
11	**KIN001-260**	IKKB	NF-κB pathway
12	**LY317615**	PKCB	Other, kinases
13	**MK-2206**	AKT1, AKT2	PI3K/MTOR signaling
14	**Navitoclax**	BCL2, BCL-XL, BCL-W	Apoptosis regulation
15	**NSC-87877**	SHP-1 (PTPN6), SHP-2 (PTPN11)	Other
16	**PD-0325901**	MEK1, MEK2	ERK MAPK signaling
17	**PD-173074**	FGFR1, FGFR2, FGFR3	RTK signaling
18	**PI-103**	PI3Kα, DAPK3, CLK4, PIM3, HIPK2	Other, kinases
19	**RDEA119**	MEK1, MEK2	ERK MAPK signaling
20	**SNX-2112**	HSP90	Protein stability and degradation
21	**TAK-715**	p38α, p38β	JNK and p38 signaling
22	**TL-2-105**	CRAF	ERK MAPK signaling
23	**WZ3105**	SRC, ROCK2, NTRK2, FLT3, IRAK1	Other
24	**XAV939**	TNKS1, TNKS2	WNT signaling
25	**YM155**	BIRC5	Apoptosis regulation

## Data Availability

The datasets generated during and/or analyzed during the current study are available from the corresponding author upon reasonable request.

## Supplementary Materials

The following supporting information can be downloaded at: https://www.mdpi.com/article/10.3390/cancers14236009/s1, Figure S1: Dose-response plots showing single-agent in vitro cytotoxicity of FK866, Docetaxel (DTX), and Cabazitaxel (CBZ) in metastatic prostate cancer cell lines. Figure S2: Dose-response plots showing FK866 + CBZ combination in metastatic PCa cell lines. Figure S3: Schematic representation of the Microfluidics-based cell Migration/Motility assay device. Figure. S4: Volcano plots representing differentially expressed genes (DEGs) following DTX, FK866 single-agent or combination treatment in human mCRPC cell lines 24 h following drug exposure (|fold-change| > 2, and *p* < 0.05). Log2 ratios are depicted in a color scale where RED represents upregulation and BLUE represents downregulation. (I) DEGs for DTX treatment for PCa cell lines. (II) DEGs for FK866 treatments for PCa cell lines. (III) DEGs for DTX+FK866 treatments for PCa cell lines. Figure S5: IPA predicted A) IFNA2, and B) mir-1-3p as top upstream regulators based on expression of genes. Figure S6: Pathway comparisons between FK866, DTX and combination (FK866 + DTX) treatments generated using Ingenuity pathway analysis predictions. Figure S7: Pathway enrichment showing FK866 treatment impacted JAK-STAT signaling pathway. Figure S8: Original Western Blots for Figure 4C. Supplementary Figure S9: Original Western Blots for Figure 9D; Table S1: List of Drugs, reagents, antibodies, and kits. Table S2: Top differentially expressed genes (DEGs) (fold change compared to untreated) following 48 h of Fk866, FK866 + DTX and DTX treatment in PC-3, PC-3M and DU145 (AI-mCRPC/NEPC) cell lines (Fold change > 2; *p* < 0.05). Table S3: Prostate cancer patient dataset details. Table S4: List the genes that were significantly dysregulated in PCa patients with BCR AND showed significant differential expression in the opposite direction following FK866 treatment in our model systems, indicating that FK866 might be capable of reversing the input signature in the patient cohort; Video S1: Microfluidics-based cell migration/motility assay.
