# Supplementary material for: Integrating Pharmacogenomics Data-Driven Computational Drug Prediction with Single-Cell RNAseq to Demonstrate the Efficacy of a NAMPT Inhibitor against Aggressive, Taxane-Resistant, and Stem-like Cells in Lethal Prostate Cancer
Source: Cancers (Basel). 2022 Dec 6;14(23):6009. doi: 10.3390/cancers14236009 (PMC9738762; doi:10.3390/cancers14236009)
Supplement: Supplementary file 1 [file cancers-14-06009-s001.zip › Supplementary Tables.pdf]

cancers

Table S1. List of Drugs, reagents, antibodies, and kits.

Reagents	Manufacturer	Location
Fetal bovine serum (FBS)	Hyclone (Thermo Fisher Scientific Inc.)	Rockford, IL, USA
Trypsin (0.25% w/v)	Hyclone (Thermo Fisher Scientific Inc.)	Rockford, IL, USA
Penicillin-Streptomycin (10,000 U/mL)	Gibco™ (Thermo Fisher Scientific Inc.)	Waltham, MA, USA
Docetaxel	Selleck Chemicals LLC	Houston, TX, USA
Cabazitaxel	Selleck Chemicals LLC	Houston, TX, USA
FK866	Selleck Chemicals LLC	Houston, TX, USA
FITC Annexin V Apoptosis Detection Kit	BD Biosciences	San Jose, CA, USA
FxCycle™ PI/RNase Staining Solution kit	Thermo Fisher Scientific Inc.	Waltham, MA, USA
RIPA lysis buffer	Thermo Fisher Scientific Inc.	Waltham, MA, USA
Protease Inhibitor Cocktail	Thermo Fisher Scientific Inc.	Waltham, MA, USA
Phosphatase Inhibitor	Thermo Fisher Scientific Inc.	Waltham, MA, USA
Pierce™ ECL Western Blotting Substrate	Thermo Fisher Scientific Inc.	Waltham, MA, USA
HES-1 specific primer Hs00172878_m1	Thermo Fisher Scientific Inc.	Waltham, MA, USA
TaqMan Gene Expression Assays	Thermo Fisher Scientific Inc.	Waltham, MA, USA
Vybrant™ DyeCycle™ Violet Stain	Thermo Fisher Scientific Inc.	Waltham, MA, USA
CM-H2DCFDA (General Oxidative Stress Indicator)	Thermo Fisher Scientific Inc.	Waltham, MA, USA
NucBlue™ Live ReadyProbes™ Reagent (Hoechst 33342)	Thermo Fisher Scientific Inc.	Waltham, MA, USA
RNeasy Plus Mini Kit	QIAGEN	Hilden, Germany
QuantiTect Reverse Transcription Kit	QIAGEN	Hilden, Germany
Quick Start Bovine Serum Albumin Standard	Bio-Rad	Hercules, CA, USA
Tris Buffer Saline (TBS)	Bio-Rad	Hercules, CA, USA
10% Tween 20	Bio-Rad	Hercules, CA, USA
Polyvinylidene fluoride membrane (PVDF)	EMD Millipore	Billerica, MA, USA
Bovine Serum Albumin (BSA)	VWR	Radnor, PA, USA
3-(4,5-dimethylthiazol-2-yl)-2,5-diphenyltetrazolium bromide (MTT)	Sigma-Aldrich Inc	St. Louis, MO, USA
Dimethyl sulfoxide (DMSO)	Sigma-Aldrich Inc	St. Louis, MO, USA
2′,7′-Dichlorofluorescin diacetate (DCFDA)	Sigma-Aldrich Inc	St. Louis, MO, USA
Bradford Reagent	Sigma-Aldrich Inc	St. Louis, MO, USA
NAD/NADH-Glo™ Assay Kit (NAMPT activity)	Promega Corp.	Madison, WI, USA
JC-1 - Mitochondrial Membrane Potential Assay Kit	Abcam	Waltham, MA, USA
NAMPT (86634S)	Cell Signaling Technology	Danvers, MA, USA

Becline-1 (3495S)	Cell Signaling Technology	Danvers, MA, USA
HES1 (11988S)	Cell Signaling Technology	Danvers, MA, USA
CD44 (3570T)	Cell Signaling Technology	Danvers, MA, USA
Cleave-caspase 9 (9505T)	Cell Signaling Technology	Danvers, MA, USA
Cleave-caspase 3 (9661T)	Cell Signaling Technology	Danvers, MA, USA
ATF-4 (11815S)	Cell Signaling Technology	Danvers, MA, USA
Anti-rabbit IgG, HRP-linked Antibody (7074S)	Cell Signaling Technology	Danvers, MA, USA
β-actin (A3854)	Sigma-Aldrich Inc	St. Louis, MO, USA

Table S2. Top differentially expressed genes (DEGs) (fold change compared to untreated) following 48 h of Fk866, FK866+DTX and DTX treatment in PC-3, PC-3M and DU145 (AI-mCRPC/NEPC) cell lines (Fold change >2; p < 0.05).

	3nM FK866		18nM DTX + 3nM FK866		18nM DTX	
**Gene**	**P value**	**Fold change**	**P value**	**Fold change**	**P value**	**Fold change**
**C1S**	6.59E-03	-7.95	NA	NA	NA	NA
**IFITM3**	3.17E-02	-6.90	NA	NA	NA	NA
**FAM229A**	7.60E-03	-5.24	4.11E-02	-3.22	NA	NA
**LHFPL5**	9.81E-03	-5.17	NA	NA	NA	NA
**DUS4L-BCAP29**	2.80E-02	-4.60	NA	NA	NA	NA
**ZACN**	2.35E-02	-4.53	NA	NA	NA	NA
**LGALS3BP**	2.39E-03	-4.28	7.15E-03	-3.18	NA	NA
**CXCL2**	4.71E-02	-3.93	NA	NA	NA	NA
**HES1**	1.62E-03	-3.84	1.54E-04	-5.96	2.24E-03	-3.29
**FAS**	2.09E-02	-3.60	NA	NA	NA	NA
**LY6G5B**	4.27E-02	-3.58	3.17E-02	-4.50	NA	NA
**CDRT4**	2.14E-02	-3.53	NA	NA	NA	NA
**MAT2A**	3.58E-02	-3.51	4.56E-02	-3.23	NA	NA
**ZC3H12A**	6.56E-03	-3.23	NA	NA	NA	NA
**IL18BP**	8.62E-03	-3.16	NA	NA	NA	NA
**PRSS53**	1.11E-02	-3.10	NA	NA	NA	NA
**PILRB**	2.08E-02	-3.06	NA	NA	NA	NA
**IGFLR1**	3.73E-02	-3.04	NA	NA	NA	NA
**ABCA1**	2.30E-02	-2.87	NA	NA	2.44E-02	-2.49
**LTB4R**	7.37E-03	-2.79	5.60E-03	-3.19	NA	NA
**UBXN11**	4.59E-04	-2.75	NA	NA	NA	NA
**NSUN5P2**	6.42E-03	-2.70	3.54E-03	-3.22	NA	NA
**RASL11A**	4.66E-02	-2.69	NA	NA	NA	NA
**RNF207**	2.01E-02	-2.64	NA	NA	NA	NA
**ZNF789**	1.62E-02	-2.61	NA	NA	NA	NA
**TERT**	3.50E-02	-2.57	NA	NA	NA	NA
**NAIP**	6.45E-03	-2.56	NA	NA	NA	NA
**MOK**	3.97E-03	-2.54	NA	NA	NA	NA
**BSCL2**	9.00E-03	-2.51	NA	NA	NA	NA
**VAMP1**	3.80E-03	-2.49	NA	NA	NA	NA
**PPIH**	2.61E-03	-2.48	NA	NA	NA	NA
**STK26**	1.62E-02	2.50	NA	NA	NA	NA
**GABARAPL1**	2.17E-02	2.50	NA	NA	NA	NA
**NCR3LG1**	1.92E-02	2.50	NA	NA	NA	NA
**RB1CC1**	4.59E-04	2.51	NA	NA	NA	NA
**CPLANE2**	1.91E-02	2.58	NA	NA	NA	NA

**TRAPPC6B**	4.88E-02	2.63	NA	NA	NA	NA
**WDR31**	8.77E-03	2.66	NA	NA	NA	NA
**TBCK**	2.76E-03	2.69	NA	NA	NA	NA
**ASNS**	5.57E-03	2.80	NA	NA	NA	NA
**OTUB2**	4.99E-03	2.87	NA	NA	NA	NA
**OTUD1**	2.10E-02	2.88	NA	NA	NA	NA
**TVP23C-CDRT4**	7.40E-03	2.90	NA	NA	NA	NA
**SYNE3**	1.50E-02	2.93	NA	NA	NA	NA
**PITPNC1**	8.92E-03	2.97	5.92E-03	3.29	NA	NA
**ADM2**	4.08E-02	2.98	NA	NA	NA	NA
**MOCOS**	3.40E-02	3.02	NA	NA	NA	NA
**CHAC1**	1.25E-03	3.18	NA	NA	NA	NA
**JPH1**	2.26E-02	3.26	NA	NA	NA	NA
**ELMOD1**	4.25E-02	3.35	NA	NA	NA	NA
**SESN3**	3.85E-02	3.40	NA	NA	NA	NA
**CYP1A1**	2.64E-02	3.72	NA	NA	NA	NA
**DDIT4**	2.17E-03	3.87	NA	NA	NA	NA
**SLC7A11**	5.16E-03	4.08	NA	NA	NA	NA
**FTCDNL1**	1.28E-02	4.12	NA	NA	NA	NA
**LAMP3**	4.16E-02	4.47	NA	NA	NA	NA
**BHLHA15**	2.14E-02	5.30	NA	NA	NA	NA
**PAPPA2**	4.78E-02	6.27	NA	NA	NA	NA
**EML5**	8.91E-03	7.23	NA	NA	NA	NA

Table S3. Prostate cancer patient dataset details.

**Sample name**	**Patient ID**	**age**	**race**	**Biochemical Recurrence_BCR**	**Gleason Score**	**pSpTage**
GSM1323647	MCC-PT081	78	W	0	7	pT3
GSM1323648	MCC-PT127	61	W	0	6	pT2
GSM1323649	MCC-PT168	63	W	0	6	pT2
GSM1323650	MCC-PT184	58	NA	0	7	pT3
GSM1323651	MCC-PT199	57	W	0	7	pT2
GSM1323652	MCC-PT236	54	W	0	7	pT2
GSM1323653	MCC-PT243	43	NA	0	7	pT2
GSM1323654	UTPC008	57	NA	0	7	pT1C
GSM1323655	UTPC020	66	NA	0	8	pT2B
GSM1323656	UTPC107	50	NA	0	6	pT1C
GSM1323657	UTPC162	69	NA	0	7	pT1C
GSM1323658	VA-PC-00-87	68.8	B	0	7	pT2C
GSM1323659	VA-PC-00-90	55.6	B	0	7	pT2
GSM1323660	VA-PC-00-91	62.1	W	0	7	pT2C

GSM1323661	VA-PC-00-92	63.1	B	0	8	pT3A
GSM1323662	VA-PC-00-93	52.5	W	0	7	pT2A
GSM1323663	VA-PC-00-94	62.4	B	0	7	pT3A
GSM1323664	VA-PC-00-95	69	B	0	7	pT2C
GSM1323665	VA-PC-00-97	51.4	W	0	6	pT2C
GSM1323666	VA-PC-00-98	68.4	W	0	7	pT2C
GSM1323667	VA-PC-91-61	56.6	W	0	7	pT2C
GSM1323668	VA-PC-91-62	63.8	W	0	8	pT2C
GSM1323669	VA-PC-91-64	69.1	W	0	7	pT2C
GSM1323670	VA-PC-92-13	64.9	W	0	7	pT2A
GSM1323671	VA-PC-92-14	70.2	W	0	7	pT2C
GSM1323672	VA-PC-92-66	65.1	W	0	7	pT2
GSM1323673	VA-PC-92-67	66.6	W	0	7	pT2
GSM1323674	VA-PC-93-19	64.1	W	0	7	pT2A
GSM1323675	VA-PC-93-68	61.3	W	0	7	pT2C
GSM1323676	VA-PC-94-28	56.3	B	0	7	pT2C
GSM1323677	VA-PC-94-70	69.6	B	0	7	pT3A
GSM1323678	VA-PC-96-45	50.4	W	0	7	pT3A
GSM1323679	VA-PC-97-47	53.7	W	0	7	pT2C
GSM1323680	VA-PC-97-48	50	B	0	7	pT2C
GSM1323681	VA-PC-97-49	64.2	W	0	6	pT2A
GSM1323682	VA-PC-97-50	60.5	W	0	7	pT2A
GSM1323683	VA-PC-97-51	57.3	B	0	7	pT2B
GSM1323684	VA-PC-97-52	64	W	0	7	pT2C
GSM1323685	VA-PC-97-74	72.4	W	0	7	pT2C
GSM1323686	VA-PC-99-54	59	W	0	7	pT2A
GSM1323687	VA-PC-99-55	47.2	B	0	7	pT2C
GSM1323688	VA-PC-99-75	54.4	W	0	7	pT2A
GSM1323689	VA-PC-99-76	54.7	W	0	7	pT2C
GSM1323690	VA-PC-99-77	55.9	B	0	6	pT2A
GSM1323691	VA-PC-99-78	62.4	B	0	7	pT2A
GSM1323692	VA-PC-99-79	64.8	W	0	7	pT2B
GSM1323693	VA-PC-99-80	54.4	B	0	7	pT2C
GSM1323694	VA-PC-99-81	65.6	W	0	7	pT2
GSM1323695	VA-PC-99-82	62.5	W	0	7	pT2C
GSM1323696	VA-PC-99-83	50.4	B	0	7	pT2A
GSM1323697	VA-PC-99-84	64.7	W	0	6	pT2A
GSM1323698	MCC-PT197	66	W	1	6	pT1C
GSM1323699	MCC-PT220	61.3	W	1	6	pT1C
GSM1323700	MCC-PT264	60.1	W	1	5	NA
GSM1323701	UTPC004	71	NA	1	9	pT1C

GSM1323702	UTPC004	71	NA	1	9	pT1C
GSM1323703	UTPC009	50	NA	1	7	pT2B
GSM1323704	UTPC009	50	NA	1	7	pT2B
GSM1323705	UTPC019	64	NA	1	7	pT2A
GSM1323706	UTPC019	64	NA	1	7	pT2A
GSM1323707	UTPC021	68	NA	1	7	pT1C
GSM1323708	UTPC021	68	NA	1	7	pT1C
GSM1323709	UTPC029	58	NA	1	7	pT2A
GSM1323710	UTPC029	58	W	1	7	pT2A
GSM1323711	UTPC034	55	NA	1	7	pT2B
GSM1323712	UTPC034	55	NA	1	7	pT2B
GSM1323713	UTPC041	71	NA	1	7	pT1C
GSM1323714	UTPC058	65	NA	1	7	pT2B
GSM1323715	UTPC088	62	NA	1	6	pT1C
GSM1323716	UTPC093	58	NA	1	7	pT2A
GSM1323717	UTPC099	67	NA	1	9	pT2B
GSM1323718	UTPC101	70	NA	1	8	pT3B
GSM1323719	UTPC104	59	NA	1	7	pT2A
GSM1323720	UTPC110	53	NA	1	9	pT2B
GSM1323721	UTPC114	60	NA	1	7	pT1C
GSM1323722	UTPC116	60	NA	1	7	pT2A
GSM1323723	UTPC127	58	NA	1	7	pT3B
GSM1323724	UTPC131	51	NA	1	7	pT3B
GSM1323725	UTPC132	65	NA	1	7	pT2A
GSM1323726	UTPC141	74	NA	1	7	pT2A
GSM1323727	UTPC146	72	W	1	8	pT3B
GSM1323728	UTPC147	66	NA	1	7	pT2A
GSM1323729	UTPC160	57	NA	1	7	pT1C
GSM1323730	UTPC164	66	NA	1	7	pT2A
GSM1323731	UTPC170	72	NA	1	7	pT1C
GSM1323732	VA-PC-00-86	59.6	B	1	7	pT3B
GSM1323733	VA-PC-00-88	53.5	B	1	7	pT2C
GSM1323734	VA-PC-00-96	58.4	W	1	7	pT2C
GSM1323735	VA-PC-00-99	65.7	B	1	7	pT3A
GSM1323736	VA-PC-90-4	59	NA	1	8	pT3B
GSM1323737	VA-PC-91-65	55	B	1	9	pT3B
GSM1323738	VA-PC-92-11	63.9	W	1	8	pT3B
GSM1323739	VA-PC-92-9	67.4	W	1	7	pT3B
GSM1323740	VA-PC-93-20	52	W	1	8	pT2C
GSM1323741	VA-PC-93-21	66.5	W	1	7	pT2C
GSM1323742	VA-PC-93-24	66.6	W	1	7	pT2

GSM1323743	VA-PC-94-71	64.8	W	1	8	pT2C
GSM1323744	VA-PC-95-34	62	W	1	7	pT2C
GSM1323745	VA-PC-95-37	57.7	B	1	7	pT2C
GSM1323746	VA-PC-95-41	58	B	1	7	pT2C
GSM1323747	VA-PC-95-42	66.8	W	1	7	pT4
GSM1323748	VA-PC-96-43	61.8	W	1	7	pT2C
GSM1323749	VA-PC-96-44	51.8	B	1	7	pT2C
GSM1323750	VA-PC-97-46	58.6	W	1	7	pT2C
GSM1323751	VA-PC-98-53	61.6	B	1	8	pT2A
GSM1323752	VA-PC-99-85	56.9	W	1	7	pT3A

Table S4. List the genes that were significantly dysregulated in PCa patients with BCR AND showed significant differential expression in the opposite direction following FK866 treatment in our model systems, indicating that FK866 might be capable of reversing the input signature in the patient cohort.

	**Patient Cohort (BCR = 1 vs BCR = 0)**				**PCa Cells lines (FK866 treatment vs baseline)**			
**Gene symbol**	**Fold change (BCR = 1 vs. BCR = 0)**	**LSMean (BCR = 1)**	**LSMean (BCR = 0)**	**P-value (BCR = 1 vs. BCR = 0)**	**Fold change (3nM FK866 vs. No treatment)**	**LSMean (3nM FK866)**	**LSMean (No treatment)**	**P-value (3nM FK866 vs. No treatment)**
**SLX1B-SULT1A4**	1.74	1.52	0.88	5.72E-03	−2.20	3.51	7.73	1.07E-02
**IFITM3**	1.73	13.19	7.64	1.45E-03	−6.90	5.53	38.15	3.17E-02
**TMEM80**	1.37	3.64	2.66	6.27E-03	−1.96	7.12	13.98	3.09E-02
**NPIPB12**	1.30	6.12	4.71	3.02E-02	−1.58	9.77	15.48	4.19E-02
**LTB4R**	1.28	4.41	3.43	2.28E-03	−2.79	5.90	16.48	7.37E-03
**ZC3H12A**	1.24	11.91	9.59	4.22E-02	−3.23	3.34	10.78	6.56E-03
**TMEM120B**	1.23	12.73	10.35	3.95E-02	−1.64	36.80	60.23	2.32E-02
**POGK**	−1.11	11.05	12.26	3.01E-02	1.44	138.76	96.57	3.42E-02
**RPS6KA5**	−1.14	9.74	11.10	4.43E-02	2.25	34.36	15.30	3.51E-02
**ZNF770**	−1.20	14.74	17.66	4.43E-02	1.75	172.40	98.49	1.57E-02
**AKAP11**	−1.21	30.41	36.90	3.32E-04	1.77	75.18	42.56	3.86E-02
**PROSER1**	−1.22	10.82	13.22	3.90E-03	1.55	65.05	41.85	4.81E-02
**FERMT2**	−1.23	11.70	14.39	4.20E-02	1.97	190.70	97.00	1.34E-02
**SPRED1**	−1.23	9.71	11.97	4.59E-02	1.83	85.88	46.93	2.26E-02
**EIF5**	−1.25	29.42	36.77	8.90E-04	1.72	829.98	482.60	2.91E-02
**TRNT1**	−1.26	17.60	22.14	3.28E-02	1.74	27.99	16.10	1.87E-02
**ARL5B**	−1.33	22.89	30.36	3.41E-03	1.77	46.36	26.20	3.67E-02
**GABARAPL1**	−1.33	14.59	19.37	8.31E-05	2.50	27.86	11.15	2.17E-02
**EML5**	−1.34	7.66	10.26	1.38E-02	7.23	41.73	5.77	8.91E-03
**FBXL4**	−1.37	12.12	16.55	1.57E-02	1.61	43.43	27.03	3.74E-02
